# Disruption of Super‐Enhancers in Activated Pancreatic Stellate Cells Facilitates Chemotherapy and Immunotherapy in Pancreatic Cancer

**DOI:** 10.1002/advs.202308637

**Published:** 2024-02-28

**Authors:** Yazhou Wang, Kai Chen, Gang Liu, Chong Du, Zhaoxia Cheng, Dan Wei, Fenfen Li, Chen Li, Yinmo Yang, Ying Zhao, Guangjun Nie

**Affiliations:** ^1^ Pancreas Center The First Affiliated Hospital of Nanjing Medical University Nanjing 210000 China; ^2^ CAS Key Laboratory for Biomedical Effects of Nanomaterials & Nanosafety CAS Center of Excellence in Nanoscience National Center for Nanoscience and Technology Beijing 100190 China; ^3^ Department of General Surgery Peking University First Hospital Beijing 100034 China; ^4^ Key Laboratory of Molecular Epigenetics of the Ministry of Education Northeast Normal University Changchun 130024 China; ^5^ Department of Oncology The Second Affiliated Hospital of Xi'an Jiaotong University Xi'an 710061 China

**Keywords:** activated pancreatic stellate cells, combination therapy, pancreatic cancer, super‐enhancer

## Abstract

One major obstacle in the drug treatment of pancreatic ductal adenocarcinoma (PDAC) is its highly fibrotic tumor microenvironment, which is replete with activated pancreatic stellate cells (a‐PSCs). These a‐PSCs generate abundant extracellular matrix and secrete various cytokines to form biophysical and biochemical barriers, impeding drug access to tumor tissues. Therefore, it is imperative to develop a strategy for reversing PSC activation and thereby removing the barriers to facilitate PDAC drug treatment. Herein, by integrating chromatin immunoprecipitation (ChIP)‐seq, Assays for Transposase‐Accessible Chromatin (ATAC)‐seq, and RNA‐seq techniques, this work reveals that super‐enhancers (SEs) promote the expression of various genes involved in PSC activation. Disruption of SE‐associated transcription with JQ1 reverses the activated phenotype of a‐PSCs and decreases stromal fibrosis in both orthotopic and patient‐derived xenograft (PDX) models. More importantly, disruption of SEs by JQ1 treatments promotes vascularization, facilitates drug delivery, and alters the immune landscape in PDAC, thereby improving the efficacies of both chemotherapy (with gemcitabine) and immunotherapy (with IL‐12). In summary, this study not only elucidates the contribution of SEs of a‐PSCs in shaping the PDAC tumor microenvironment but also highlights that targeting SEs in a‐PSCs may become a gate‐opening strategy that benefits PDAC drug therapy by removing stromal barriers.

## Graphical abstract

This study identifies the landscape of super‐enhancers (SEs) in activated pancreatic stellate cells (PSCs). Many activation‐associated genes are regulated by SEs, with IL6 and FAP‐α serving as proof of concept to demonstrate the regulation of SEs in activated PSCs. Disrupting SEs in activated PSCs alleviates fibrosis and enhances chemotherapy and immunotherapy in pancreatic cancer.

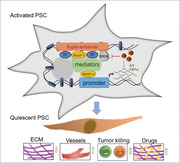

## 1 Introduction

Pancreatic ductal adenocarcinoma (PDAC) stands as one of the most formidable malignant tumors, characterized by an alarmingly low 5‐year survival rate of just 11%.^[^ ^1^ ^]^ PDAC is mostly asymptomatic at early stages, and the lesions are always unresectable when detected.^[^ ^2^ ^]^ Therefore, anti‐cancer drugs remain the mainstay strategy to improve PDAC patients’ survival. However, the highly fibrotic tumor microenvironment constitutes a biophysical and biochemical barrier that hinders the drug treatment of PDAC.^[^ ^3^ ^]^ Activated pancreatic stellate cells (a‐PSCs) play essential roles in shaping the fibrotic tumor microenvironment of PDAC via generating abundant extracellular matrix and secreting various cytokines. Many studies aimed at clearing the extracellular matrix (ECM) produced by a‐PSCs or breaking the crosstalk between a‐PSCs and pancreatic cancer cells have been conducted to enhance drug treatment efficacy. Still, the outcomes are disappointing.^[^ ^4^ ^]^ In recent years, researchers have sought to reverse PSCs' activation into a quiescent‐like phenotype, thereby fundamentally altering their functions.^[^ ^5^ ^]^ For example, all‐trans retinoic acid (ATRA) has entered a phase I clinical trial as a stromal targeting agent for pancreatic cancer due to its demonstrated capacity to reprogram a‐PSCs.^[^ ^6^ ^]^ However, the drug exhibited limited efficacy in improving pancreatic cancer patients’ survival and failed to be approved for clinical use. Therefore, it is imperative to develop a novel strategy for reversing the activation of PSCs and thereby removing the barriers to facilitate PDAC drug treatment.

Super‐enhancers (SEs) refer to a cluster of transcriptional enhancers characterized by abundant histone 3 lysine 27 acetylation (H3K27ac) marks that drive the transcription of critical genes defining cell identity.^[^ ^7^ ^]^ A previous study has shown that hepatocellular carcinoma cells possess an aberrant SE landscape; another investigation on multiple myeloma suggested that targeting SEs may represent a novel therapeutic strategy against cancers.^[^ ^8^ ^]^ These studies confirm that SEs can contribute to tumor cell development. However, whether SEs are implicated in the formation of tumor microenvironment remains largely unknown. Given that the activation of PSCs necessitates the simultaneous overexpression of various genes related to fibrogenesis, we hypothesize that SEs play essential roles during this process and that disruption of SEs holds the promise of reversing a‐PSCs into a quiescent‐like phenotype.

To test these hypotheses and further explore the clinical significance of disrupting SEs in the treatment of PDAC, we herein characterized the super‐enhancer (SE) landscape within a‐PSCs using a combination of chromatin immunoprecipitation (ChIP)‐seq, Assays for Transposase‐Accessible Chromatin (ATAC)‐seq, and RNA‐seq techniques. Interestingly, we found that SEs of a‐PSCs drive the expression of multiple genes participating in PSC activation. These findings prompted us to try to reverse the activation of a‐PSCs with JQ1, a well‐established BET inhibitor known for its efficacy in suppressing SE‐mediated transcription in cancer cells.^[^ ^8^, ^9^ ^]^ Encouragingly, JQ1 treatments reversed the activation of a‐PSCs and decreased stromal fibrosis in orthotopic and patient‐derived xenograft (PDX) models. More importantly, the disruption of SEs by JQ1 treatments enhanced vascularization, facilitated drug delivery, and changed the immune landscape in PDAC, thereby improving the efficacies of both chemotherapy (with gemcitabine) and immunotherapy (with IL‐12) in the aforementioned in vivo models.

Our research clarifies the contribution of SEs to the formation of PDAC tumor microenvironment and highlights that targeting SEs in a‐PSCs may represent a gate‐opening strategy that promotes PDAC drug therapy by eliminating tumor stromal barriers.

## 2 Results

### 2.1 Activated PSCs Played A Major Part in Shaping the Fibrotic Microenvironment in PDAC Tissues

**Figure 1:**
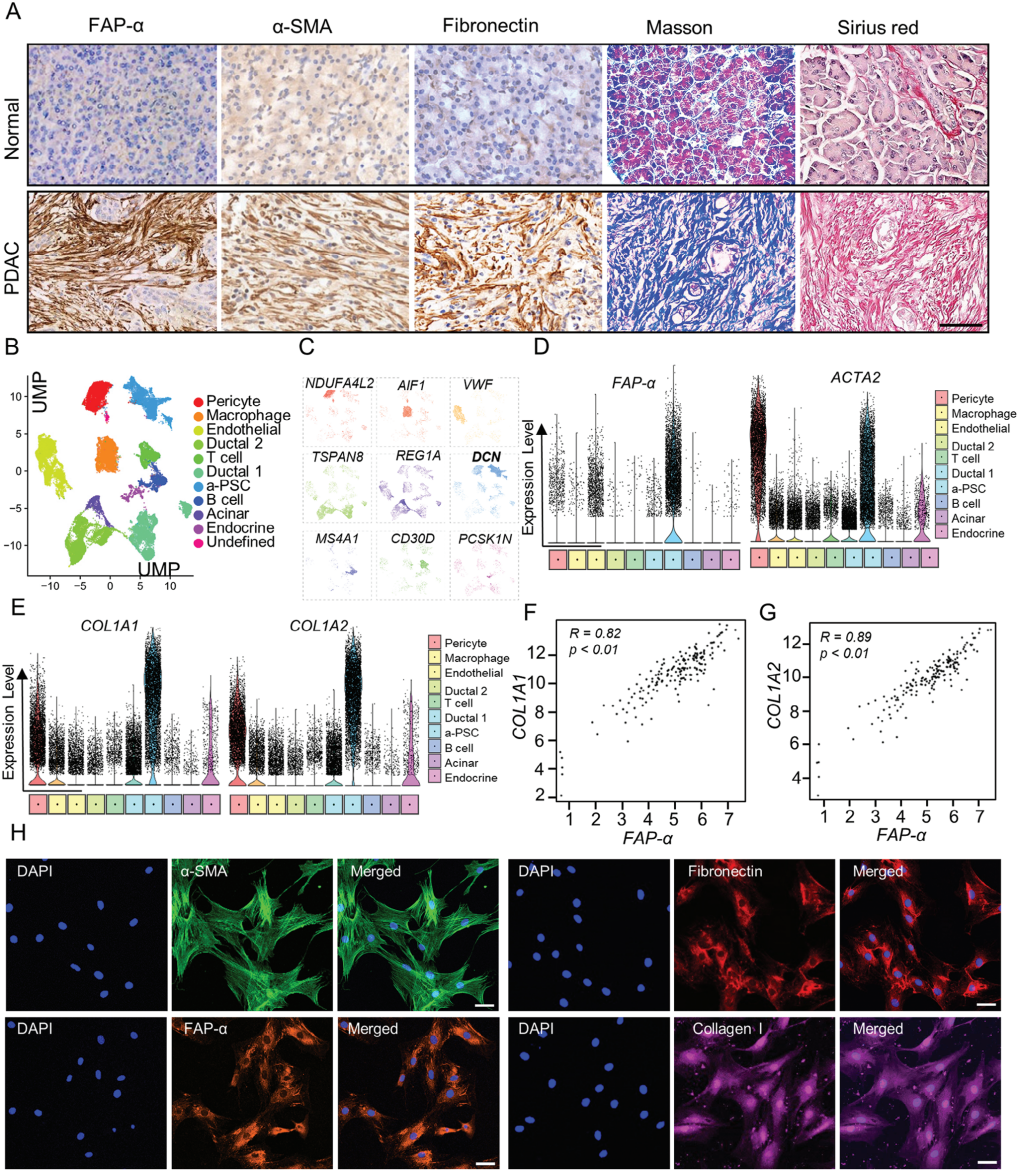
Activated PSCs played a major role in generating the fibrotic stroma of PDAC tissues. A) Activated PSCs (a‐PSCs) and collagen deposition in normal pancreas and PDAC tissues detected by IHC staining for FAP‐α, α‐SMA, and fibronectin, Masson's trichrome staining, and Sirius Red staining. Scale bar, 50 µm, *n =* 62. B) The uMAP plot shows the cell clusters from 24 PDAC and 11 normal pancreatic tissues. Cell types are coded with different colors. C) Marker genes for different cell clusters. D) Violin plots showing the normalized expression levels of FAP‐α and ACTA2 across cell subpopulations. E) Violin plots showing the normalized expression levels of *COL1A1* and *COL1A2* across cell subpopulations. F) The correlation between the expression levels of *FAP‐α* and *COL1A1* in PDAC tissues (data from TCGA). G) The correlation between the expression levels of *FAP‐α* and *COL1A2* in PDAC tissues (data from TCGA). H) Immunofluorescence assays showing the protein levels of typical activation markers FAP‐α, α‐SMA, fibronectin, and collagen I in human primary a‐PSCs. Scale bar, 50 µm, *n =* 3.

Desmoplasia is recognized as the most characteristic histopathological feature of PDAC, during which a‐PSCs play essential roles by producing a substantial amount of stromal components.^[^ ^10^ ^]^ To test whether a‐PSCs and ECM constitute the major part of PDAC tissues, we first analyzed the expression levels of a‐PSCs markers FAP‐α, α‐SMA, and fibronectin by immunohistochemistry (IHC) and appraised collagen deposition by Masson's trichrome staining and Sirius Red staining in PDAC tissues obtained from patients diagnosed with PDAC pathologically. While paired normal pancreas tissues displayed minimal expression of FAP‐α, α‐SMA, and fibronectin and negligible collagen deposition, PDAC tissues exhibited overwhelmingly high levels of FAP‐α, α‐SMA and fibronectin expression, and collagen deposition (**Figure** **1**A, Figure S1A, Supporting Information). These results verified that a‐PSCs and ECM constitute the major part of PDAC tissues. We conducted additional analysis of the correlation between patient survival and expression levels of activation‐associated genes using data from the TCGA database. The findings revealed that patients with elevated expression of activation‐associated genes (*FAP‐α, COL1A2, COL4A1*) exhibited significantly shorter disease‐free survival (DFS) time (Figure S1B–D, Supporting Information).

To further verify whether a‐PSCs are the primary cellular source of ECM in PDAC tissues, we analyzed the single‐cell sequencing data available in the GSA database (Genome Sequence Archive).^[^ ^11^ ^]^ As shown in the uMAP plot of Figure 1B, 11 distinct cellular populations have been identified in PDAC tissues based on well‐established cell type‐specific genes (namely the marker genes) documented in the literature.^[^ ^12^ ^]^ In the heatmap of Figure S2A (Supporting Information), the 5 marker genes with the highest expression levels in each cell population are displayed, among which one signature gene was designated for each cell population (Figure 1C). The cell population corresponding to a‐PSCs is colored sky blue in Figure 1B, and DCN was designated as the signature gene for this cell population by previous research (Figure 1C).^[^ ^13^ ^]^ FAP‐α and ACTA2 are well‐established marker genes for a‐PSCs. To verify the identity of the a‐PSCs population determined based on the single‐cell sequencing data, we characterized the expression levels of FAP‐α and ACTA2 across the cell populations and summarized the findings in violin plots in Figure 1D. According to our analysis, compared with the other cell populations, the a‐PSCs population exhibited markedly higher expression levels of these two genes, especially FAP‐α (Figure 1D), thereby confirming the identity of the cell population. Notably, according to our gene ontology (GO) analysis, the marker genes for this cell population were enriched with GO terms “collagen structure” and “extracellular matrix” (Figure S2B, Supporting Information). Additionally, this a‐PSCs population expressed higher levels of collagen‐associated genes, such as *COL1A1* and *COL1A2*, compared with other cell clusters (Figure 1E). Moreover, based on data from The Cancer Genome Atlas (TCGA) database, the expression level of FAP‐α exhibited strong positive correlations with those of *COL1A1*, *COL1A2*, *COL4A1*, *COL4A2*, *FN1*, and *DCN* genes (Figure 1F,G; Figure S2C, Supporting Information). These data indicated that a‐PSCs were the primary cellular source of ECM in PDAC tissues and that these cells played a major part in shaping the fibrotic tumor microenvironment. Therefore, targeting these cells may reshape the PDAC microenvironment, thereby benefiting PDAC drug treatment.

### 2.2 Isolation of Primary Human a‐PSCs from PDAC Tissues and Verification of Their Identity

We utilized primary human a‐PSCs as the cellular model in this study. These cells were isolated from PDAC tissues obtained from patients diagnosed with PDAC using the “outgrowth” method described previously.^[^ ^14^ ^]^ To confirm their identity, the isolated cells were then characterized by immunofluorescence (IF) staining for a‐PSC marker proteins α‐SMA, FAP‐α, fibronectin, and collagen I. Representative IF findings depicted in Figure 1H indicated high expression levels of these marker proteins, confirming that the isolated cells were indeed a‐PSCs. These a‐PSCs were used in the subsequent experiments.

### 2.3 Super‐Enhancers (SEs) Contributed Significantly to the Activation of Human a‐PSCs

**Figure 2:**
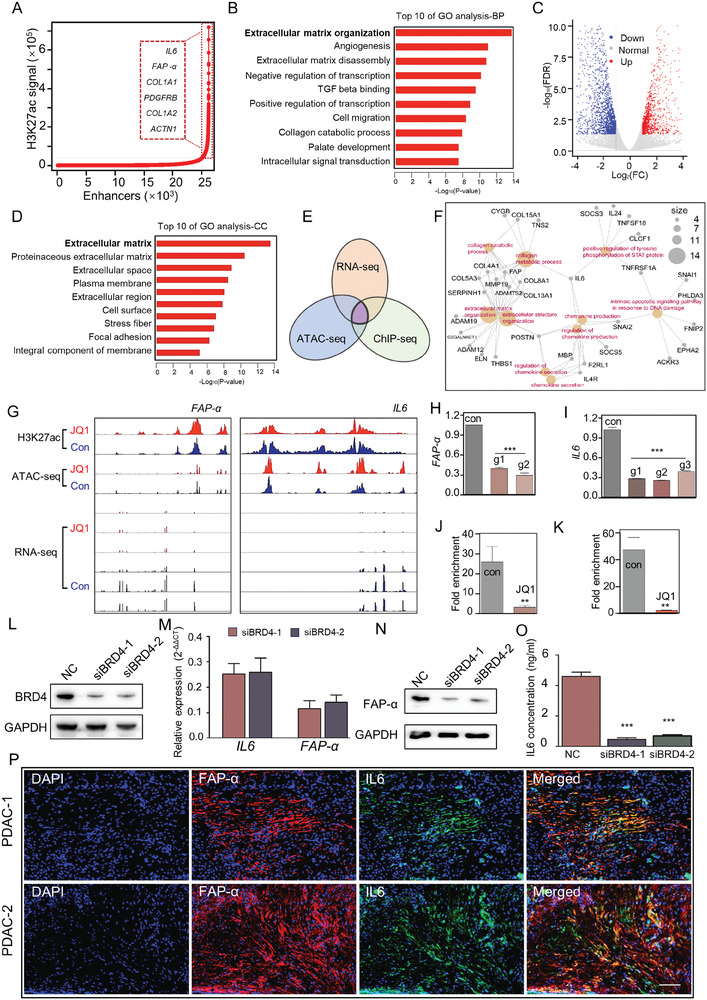
Super‐enhancers were associated with genes involved in a‐PSCs activation. A) SE regions in a‐PSCs were plotted according to H3K27Ac ChIP‐seq data. Enhancers above the inflection point of the curve (those in the dotted rectangle of the curve) were defined as SEs. Representative SE‐associated genes are shown in a dotted rectangle on the left of the curve. B) The top 10 most significant GO terms in the biological process category enriched by the SE‐associated genes in a‐PSCs. C) A volcano plot displaying the differentially expressed genes (DEGs) between the control (con) and JQ1 groups of a‐PSCs as determined by RNA‐seq. D) The top 10 most significant GO terms in the cellular component category enriched by the down‐regulated DEGs. E) A Venn diagram displaying the overlap between the down‐regulated DEGs and SE‐associated genes in a‐PSCs identified by both H3K27Ac ChIP‐seq and ATAC‐seq techniques. F) The genes that fell into the overlapping part in the Venn diagram of panel E) are summarized into a network. G) H3K27Ac ChIP‐seq, ATAC‐seq, and RNA‐seq profiles for FAP‐α and IL6 are visualized by the IGV software. H,I) Relative mRNA expression levels of FAP‐α and IL6 after knocking out multiple enhancer sites with the CRISPR/Cas9 technique. J,K) Decreased occupancy of BRD4 in the *FAP‐α* and *IL6* SE regions after treatment with JQ1. L) Western blotting analysis for BRD4 protein levels in a‐PSCs after the cells were transfected with siRNAs against BRD4 or the negative control (NC). M) Relative *FAP‐α* and *IL6* mRNA expression levels in a‐PSCs transfected with siRNAs against BRD4 or the NC. N) Western blotting analysis for FAP‐α protein levels in a‐PSCs transfected with siRNAs against BRD4 or the NC. O) Concentrations of IL6 in the cell culture media of a‐PSCs transfected with siRNAs against BRD4 or the NC. P) Immunofluorescence assays for FAP‐α and IL6 in two representative PDAC tissues, *n =* 6. Scale bar, 100 µm. Data are represented as mean ± standard deviation (SD) of three independent experiments or triplicates, and *p* values were determined by a two‐tailed unpaired *t*‐test. ** *p* < 0.01, *** *p* < 0.001 compared with the con or NC groups.

SEs are genomic elements highly enriched with H3K27ac marks, which have been proven to regulate the expression of crucial cell identity‐determining genes and have been targeted in treating several diseases.^[^ ^15^ ^]^ Given that the activation of PSCs requires the simultaneous overexpression of multiple fibrogenesis‐associated genes, we hypothesize that SEs exert crucial functions during this process. To test this hypothesis, we performed H3K27ac ChIP‐seq to characterize the SE landscape in a‐PSCs. The enhancer regions captured by H3K27ac ChIP‐seq were stitched together and ranked according to their signal intensity to form a signal intensity curve (**Figure** **2**A). Enhancers with a signal intensity above the inflection point on the signal intensity curve were considered SEs, while those with a signal intensity below the inflection point were considered typical enhancers (TEs) (Figure 2A; Figure S3A, Supporting Information). These SEs in a‐PSCs can potentially regulate the expression of 1121 genes (hereinafter referred to as SE‐associated genes) (Figure S3B, Supporting Information), among which several representative ones are summarized in Figure 2A. The representative genes were then subjected to a GO analysis, which revealed that these genes were mainly enriched in biological process (BP) terms associated with ECM organization (Figure 2B), implying that SEs may contribute to the activation of PSCs. To further verify these results, we performed ATAC‐seq in the a‐PSCs and compared the SE profiles obtained by ChIP‐seq and ATAC‐seq visually through Integrative Genomic Visualization (IGV) software at the whole‐genome level. We found that the SE profiles acquired by both techniques were nearly identical (Figure S3C, Supporting Information), thereby confirming the reliability of our H3K27ac ChIP‐seq results.

To test whether SEs in a‐PSCs can indeed regulate the expression levels of ECM organization‐related genes, we treated the isolated primary human PSCs with JQ1, a BET inhibitor proved to disrupt SE‐mediated transcriptional regulation by depleting the occupancy of BRD4 at SE regions.^[^ ^8^, ^15^, ^16^ ^]^ The JQ1‐treated a‐PSCs were first subjected to H3K27ac ChIP‐seq and ATAC‐seq analyses to determine if JQ1 can directly alter the SE profile and chromatin accessibility in these cells. These analyses revealed no significant differences in SE profile and chromatin accessibility between JQ1‐treated and untreated a‐PSCs (Figure S4A–D, Supporting Information). Moreover, the top 10 GO terms enriched by SE‐associated genes exhibited no apparent diversity (Figure S4E,F, Supporting Information). These results showed that JQ1 treatment did not alter SE landscape or chromatin accessibility in a‐PSCs. However, as revealed by our RNA‐seq analysis, the JQ1 treatment did markedly alter the expression levels of numerous genes in a‐PSCs (Figure 2C), among which many significantly downregulated ones were enriched with ECM‐related cellular component terms in our GO analysis (Figure 2D), revealing a significant inhibitory effect of JQ1 on genes associated with PSCs activation. Furthermore, by intersecting SE‐associated genes identified by H3K27ac ChIP‐seq and ATAC‐seq techniques with the differentially expressed genes (DEGs) identified by RNA‐seq, we found that the SE‐associated genes significantly downregulated by JQ1 treatment were predominantly enriched with ECM generation and chemokine secretion‐related pathways (Figure 2E,F). Taken together, these data indicated that SE‐mediated aberrant overexpression of genes associated with ECM generation and chemokine secretion contributed significantly to the activation of human a‐PSCs.

Notably, several marker genes for a‐PSCs, namely *FAP‐α*, *IL6*, *ACTN1*, *ACTA2*, *COL1A1*, and *COL4A1*, were among the SE‐associated genes significantly downregulated by JQ1 treatment (Figure 2G, Figure S5, Supporting Information). Therefore, as a proof‐of‐concept, we took *FAP‐α* and *IL6* as representative genes to verify the regulatory effect of JQ1 treatment on SE‐associated genes in a‐PSCs. Based on the H3K27ac ChIP‐seq data, we removed multiple enhancer sites in the SE regions of *FAP‐α* and *IL6* using the CRISPR/Cas9 technique. The mRNA levels of both genes were significantly reduced after removing the enhancer sites (Figure 2H,I). These findings indicated that the transcription of these two genes was indeed activated by the SEs. Given that JQ1 treatment could significantly decrease the expression levels of *FAP‐α* and *IL6*, we hypothesize that BRD4, an essential transcription regulator whose occupancy in SE regions can be disrupted by JQ1, is crucial for SE‐mediated overexpression of these two genes. To test this hypothesis, we conducted ChIP‐qPCR assays in JQ1‐treated and untreated a‐PSCs. The results clearly demonstrated that JQ1 treatment markedly abolished the occupancy of BRD4 at the SE regions of FAP‐α and IL6 (Figure 2J,K). Furthermore, siRNA‐mediated BRD4 knockdown significantly decreased mRNA and protein levels of FAP‐α and IL6 in a‐PSCs (Figure 2L–O). These results demonstrated that BRD4 is indeed crucial for SE‐mediated overexpression of FAP‐α and IL6, thereby clarifying the molecular mechanism underlying JQ1's inhibitory effects on the transcription of specific SE‐associated genes. To confirm the overexpression of FAP‐α and IL6 in activated pancreatic stellate cells, we conducted an immunofluorescence assay using PDAC tissues. As depicted in Figure 2P, FAP‐α and IL6 demonstrated substantial upregulation and were predominantly localized in a‐PSCs within the PDAC tissues. This reinforced the significant role of these two markers in identifying a‐PSCs.

### 2.4 JQ1 Treatment Reversed the Activated Phenotype of Primary Human a‐PSCs In Vitro and In Vivo

**Figure 3:**
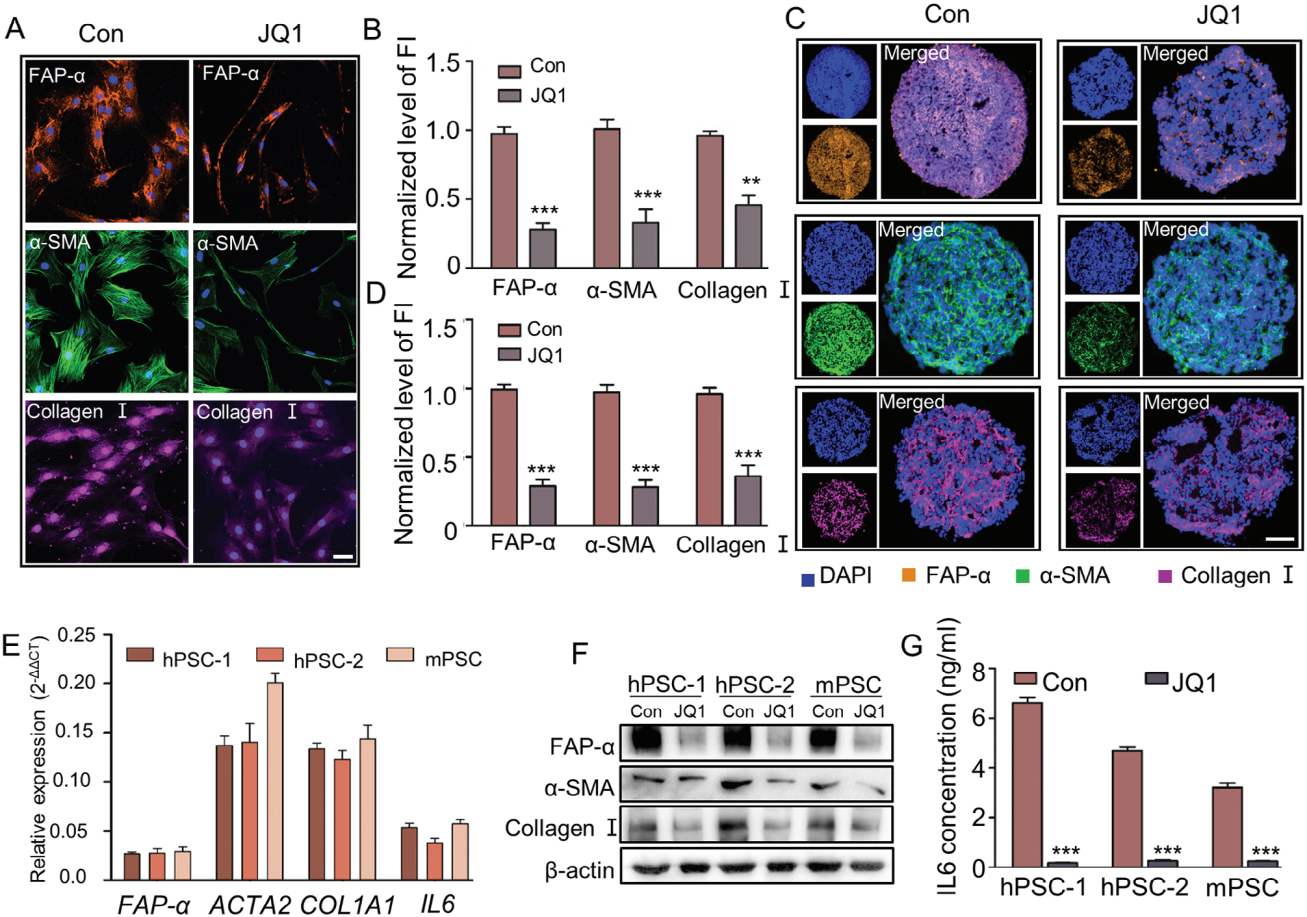
Disruption of SEs decreased the expression of genes involved in a‐PSC activation at both mRNA and protein levels. A) Immunofluorescence assays for FAP‐α, α‐SMA, and collagen I in PSCs before and after JQ1 treatment. Scale bar, 50 µm. B) Quantitative analysis of normalized fluorescence intensity (FI) levels for FAP‐α, α‐SMA, and collagen I. C) Immunofluorescence assays for FAP‐α, α‐SMA, and collagen I in slices of tumor spheres from 3D cell culture models before and after JQ1 treatment. Scale bar, 100 µm. D) Quantitative analysis of normalized fluorescence intensity (FI) levels for FAP‐α, α‐SMA, and collagen I. E) Relative mRNA expression levels of representative SE‐associated genes in human and mouse PSCs after JQ1 treatment. F) Western blotting analysis for FAP‐α, α‐SMA, and collagen I protein levels in human and mouse PSCs before and after JQ1 treatment. G) Concentrations of IL6 in the cell culture media of human and mouse PSCs before and after JQ1 treatment. Data are represented as mean ± standard deviation (SD) of three independent experiments or triplicates, and *p* values were determined by a two‐tailed unpaired *t*‐test. ** *p* < 0.01, *** *p* < 0.001 compared with the Con groups.

Prompted by the inhibitory effects of JQ1 treatment on SE‐mediated overexpression of PSCs activation‐related genes, we sought to reverse the activated phenotype of human primary a‐PSCs with JQ1. First, we evaluated the expression levels of a‐PSC marker proteins FAP‐α, α‐SMA, and collagen I and the cell morphology with IF assays. The results showed that the expression levels of FAP‐α, α‐SMA, and collagen I were significantly downregulated and that the cell morphology was markedly changed from the typical polygonal shape to an irregular spindle shape (**Figure** **3**A,B). Similar changes in marker protein abundance and cell morphology were observed in mouse PSCs (mPSCs) extracted using a typical density gradient centrifugation method after JQ1 treatment (Figure S6A,B, Supporting Information).^[^ ^14b^ ^]^ Then we tried to verify these findings in an in vitro 3D cell culturing model that mimics the PDAC tumor microenvironment, which was established by mixing PANC‐1 pancreatic cancer cells with primary human a‐PSCs to form tumor spheres. After JQ1 treatment, the spheres were subjected to IF assays. The results showed significantly decreased levels of FAP‐α, α‐SMA, and collagen I in the tumor spheres (Figure 3C,D) after JQ1 treatment. Next, we further verified the effects of JQ1 treatment on the expression levels of PSC activation‐related genes in three pancreatic stellate cell lines, including two derived from human PDAC tissues and one from mouse pancreas. The expression levels of PSC activation‐associated genes, namely *FAP‐α*, *ACTA2*, *COL1A1*, and *IL6*, were assessed by quantitative real‐time PCR (qRT‐PCR). As exhibited in Figure 3E, the expression levels of all these genes were significantly decreased by JQ1 treatment. Furthermore, the protein levels of these genes and IL6 secretion were also evidently reduced by JQ1 treatment in the three pancreatic stellate cell lines (Figure 3F,G). These data provided compelling evidence that JQ1 effectively reversed the activation of PSCs in vitro.

**Figure 4:**
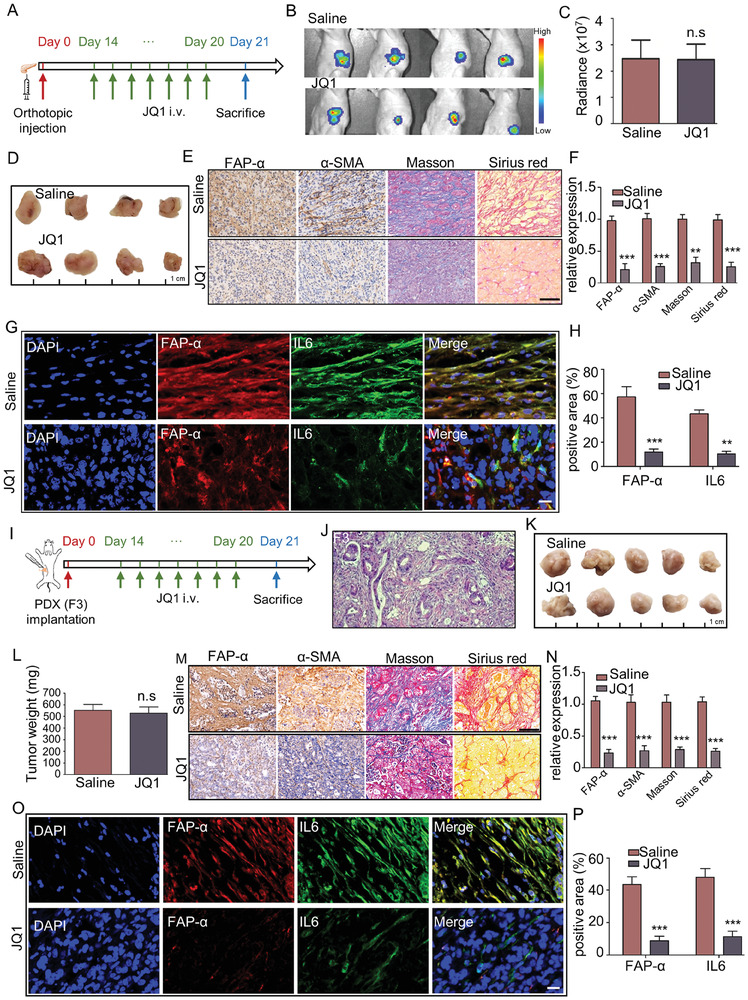
Disruption of SEs by JQ1 treatment alleviated stromal fibrosis in PDAC tissues in vivo. A) Scheme of JQ1 treatment in an orthotopic pancreatic cancer mouse model. i.v., intravenous. B) Bioluminescence images for the orthotopic tumor tissues in the con and JQ1 groups of mice on Day 21 of the experiment, *n =* 4 for each group. C) Radiance of the orthotopic tumor tissues in the con and JQ1 groups of mice. D) Pictures of the orthotopic tumor tissues obtained from the con and JQ1 groups of mice after the treatment. *n =* 4 for each group, n.s, no significance. E) IHC staining for FAP‐α and α‐SMA, Masson's trichrome staining, and Sirius Red staining analyses of the orthotopic tumor tissues obtained from the con and JQ1 groups of mice. Scale bar, 100 µm. F) Quantification of the signal intensities for the staining analyses displayed in panel (E). G) Immunofluorescence assays for IL6 and FAP‐α in slices of the orthotopic tumor tissues obtained from the con and JQ1 groups of mice. Scale bar, 20 µm. H) Quantitative analysis of immunofluorescence staining for IL6 and FAP‐α in orthotopic models. I) Scheme of JQ1 treatment in a PDX pancreatic cancer mouse model. J) A representative H&E staining finding for PDX tumor (F3) slices. K) Tumors dissected from the PDX model mice treated with saline or JQ1. *n =* 5 for each group. L) Weights of tumors dissected from the PDX model mice treated with saline or JQ1. n.s, no significance. M) IHC staining for FAP‐α and α‐SMA, Masson's trichrome staining, and Sirius Red staining analyses of the tumors obtained from the PDX model mice treated with saline or JQ1. Scale bar, 100 µm. N) Quantification of the signal intensities for the staining analyses displayed in panel (M). O) Immunofluorescence assays for IL6 and FAP‐α in slices of the tumors obtained from the PDX model mice treated with saline or JQ1. Scale bar, 20 µm. P) Quantitative analysis of immunofluorescence staining for IL6 and FAP‐α in PDX models. Data are shown as mean ± SD and *p* values were determined by a two‐tailed unpaired *t*‐test. ** *p* < 0.01, *** *p* < 0.001, n.s (not significant), *p* > 0.05 compared with the con groups.

To test whether JQ1 treatment could reverse the activation of PSCs in vivo, we constructed an orthotopic pancreatic cancer model by injecting primary human a‐PSCs and PANC‐1‐luc pancreatic cancer cells at a ratio of 1:2 into the pancreas of nude mice as previously described.^[^ ^5a^ ^]^ Two weeks later, the mice were treated with JQ1 once a day (5 mg k^−1^g), or with the same volume of saline for the control group, for 7 days (**Figure** **4**A). At the end of the treatment, the fluorescence signal of orthotopic tumors was detected with an IVIS small animal live imaging system. The tumor tissues were also excised for other analyses. There were no significant differences in fluorescence signal intensity and tumor size between the saline and JQ1 treatment groups (Figure 4B–D). However, the excised tumor tissues in the JQ1 group exhibited reduced protein levels of FAP‐α, α‐SMA, and IL6, as well as markedly decreased collagen deposition, as revealed by IHC, Masson's trichrome staining, Sirius Red staining, and IF assays (Figure 4E–H). To verify these findings obtained from the orthotopic pancreatic cancer model, we established a patient‐derived xenograft (PDX) mouse pancreatic cancer model and applied JQ1 treatment following the scheme presented in Figure 4I. At the end of the treatment, all subcutaneous xenografts were excised and analyzed. We first performed hematoxylin and eosin (H&E) staining to confirm that the xenografts were indeed PDAC (Figure 4J). Similar to the findings obtained in the orthotopic pancreatic cancer model, although there were no statistically significant differences in tumor size and weight between the saline and JQ1 groups (Figure 4K,L), the JQ1 treatment reduced FAP‐α, α‐SMA, and IL6 protein levels and collagen deposition in the xenografts (Figure 4M–P). Taken together, these results strongly support that although the administered dosage of JQ1 had no significant inhibitory effects on tumor growth, it effectively reversed the activation phenotype of a‐PSCs and alleviated fibrosis of PDAC tumors in orthotopic and PDX pancreatic cancer models.

### 2.5 JQ1 Treatment Enhanced Drug Penetration and Vascularization in PDAC Tumors

**Figure 5:**
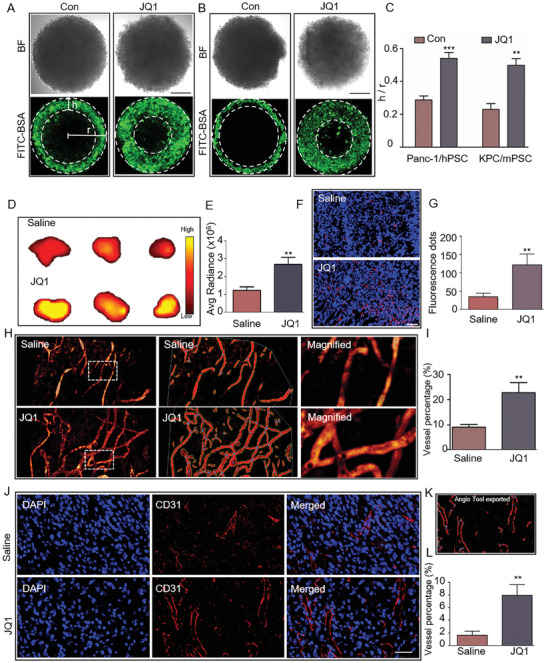
Disruption of SEs by JQ1 treatment enhanced drug penetration and promoted vascularization in PDAC tumors. A,B) Representative images showing the penetration depth of FITC‐BSA into tumor spheres from human and mouse 3D cell culture models before and after JQ1 treatment. BF, bright field. Scale bar, 100 µm. C) Quantitative analysis of the findings shown in panel (A and B). Data are represented as mean ± standard deviation (SD) of three independent experiments or triplicates, and *p* values were determined by a two‐tailed unpaired *t*‐test. D) Fluorescence signal intensities of dissected orthotopic tumors from saline‐ or JQ1‐treated mice injected with the fluorescent molecule Cy7. *n =* 3 for each group. E) Quantitative analysis of the findings shown in panel (D). F) Fluorescence signals (red dots) detected in orthotopic tumor slices from saline‐ or JQ1‐treated mice injected with 400‐nm fluorescent spheres. *n =* 3 for each group. Scale bar, 50 µm. G) Quantitative analysis of the findings shown in panel (F). H) Multi‐photon fluorescence microscopy images displaying intratumor vessels after i.v. administration of 70‐kDa FITC‐dextran into saline‐ or JQ1‐treated mice. *n =* 3 for each group. I) Quantitative analysis of tumor vessel densities in orthotopic tumors obtained from saline‐ or JQ1‐treated mice injected with the 70‐kDa FITC‐dextran. J) Representative IF images for CD31 in orthotopic tumors obtained from saline‐ or JQ1‐treated mice. Scale bar, 50 µm. *n =* 3 for each group. K) A picture exported from AngioTool showing CD31‐positive areas in an orthotopic tumor slice. L) Quantitative analysis of CD31‐positive areas in orthotopic tumors obtained from saline‐ or JQ1‐treated mice. Data are shown as mean ± SD and *p* values were determined by a two‐tailed unpaired *t*‐test. ** *p* < 0.01, *** *p* < 0.001 compared with the con or Saline groups.

Activated PSCs and ECM generated by them have been proven to hinder drug penetration into PDAC tissues. Given that JQ1 treatment could reverse PSC activation and reduce fibrosis in PDAC tissues, we first tested whether the treatment could benefit drug penetration with the in vitro 3D cell culturing models mentioned above. The tumor spheres were first treated with JQ1. After that, the tumor spheres were incubated with FITC‐conjugated bovine serum albumin (FITC‐BSA, 1 mg mL^−1^) to evaluate drug penetration with a fluorescence microscope. As shown in **Figure** **5**A–C, JQ1 treatment significantly increased the penetration depth of FITC‐BSA into tumor spheres in both human and mouse 3D cell culturing models. Then, we tried to verify these findings in vivo with the orthotopic pancreatic cancer model described above. After JQ1 treatment, Cy7 fluorescent molecules and fluorescent spheres with a diameter of 400 nm were injected into the tail vein of mice. Twenty hours later, orthotopic tumor samples were prepared, and fluorescence signals were detected with the IVIS system for Cy7 fluorescent molecules and the fluorescence microscope for fluorescent spheres. As shown in Figure 5D–G, the JQ1 treatment enhanced Cy7 signal intensity and fluorescent sphere infiltration in the orthotopic tumor tissues. These results indicated that JQ1 treatment significantly enhanced the penetration of drugs into PDAC tissues in vivo in a size‐independent manner. Taken together, our in vitro and in vivo findings demonstrate that JQ1 treatment could benefit drug penetration into PDAC tissues.

Intratumor vessels constitute the major pathway for the delivery of drugs into tumors. In PDAC tissues, the highly fibrotic stroma gives rise to a hypovascular tumor microenvironment that impedes drug delivery.^[^ ^17^ ^]^ Additionally, there is a negative correlation between fibrosis degree and vascular density in these tissues, whereby higher levels of fibrosis are associated with lower vascular density and increased vascular compression. As depicted in Figure S7A (Supporting Information), the PDAC tissues characterized by abundant stroma exhibited sparse blood vessels (dotted rectangle 1), whereas the tissues with a low degree of fibrosis displayed a dense network of blood vessels (dotted rectangle 4) (data from the Human Protein Atlas database). Therefore, we tested whether JQ1 treatment could enhance intratumor vascularization by alleviating PDAC stromal fibrosis in the orthotopic model. The tumor‐burdened mice were treated with JQ1, and their intratumor vessels were observed in vivo via multiphoton fluorescence microscopy and quantitatively analyzed by AngioTool. As shown in Figure 5H,I, a higher density of intratumor vessels was observed in the JQ1 group relative to the saline group (Figure 5I). Then, the orthotopic tumors were excised and analyzed for CD31 positivity by IF assays. CD31 is a blood vessel marker whose expression level can reflect the vascularization status. As displayed in Figure 5J–L, JQ1 treatment significantly increased the vascularization in PDAC tumors. Similar findings on CD31 positivity were also observed in the PDX model (Figure S7B–D, Supporting Information). Taken together, these results conclusively demonstrated that JQ1 effectively promoted the vascularization of PDAC tissues in vivo.

### 2.6 JQ1 Treatment Enhanced the Antitumor Efficacy of Gemcitabine (GEM) Against PDAC In Vivo

**Figure 6:**
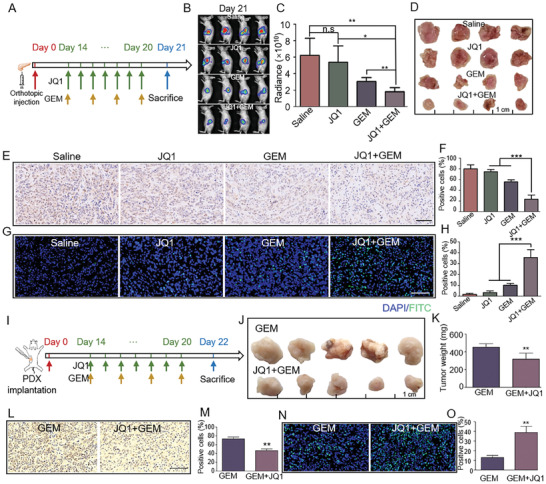
JQ1 treatment enhanced the antitumor efficacy of gemcitabine (GEM) against PDAC in vivo. A) Scheme of JQ1 and GEM treatments (or their combination) in the orthotopic pancreatic cancer mouse model. B) Bioluminescence images for the orthotopic tumor tissues in the Saline, JQ1, GEM, and JQ1+GEM groups of mice on Day 21 of the experiment, *n =* 4 for each group. C) Radiance of the orthotopic tumor tissues in the Saline, JQ1, GEM, and JQ1+GEM groups of mice. D) Pictures of excised orthotopic tumors obtained from the Saline, JQ1, GEM, and JQ1+GEM groups of mice. E) IHC staining for PCNA in orthotopic tumor slices from the Saline, JQ1, GEM, and JQ1+GEM groups of mice. F) Quantitative analysis of the percentages of PCNA‐positive cells based on the findings presented in panel (E). G) TUNEL staining of the orthotopic tumor slices. H) Quantitative analysis of the percentages of TUNEL‐positive apoptotic tumor cells based on the findings presented in panel (G). I) Scheme of JQ1 and GEM treatments (or their combination) in the PDX pancreatic cancer mouse model. J) Pictures of excised PDX tumors obtained from the GEM and JQ1+GEM groups of mice, *n =* 5 for each group. K) Weights of the excised PDX tumors. L) IHC staining for PCNA in PDX tumor slices from the GEM and JQ1+GEM groups of mice. M) Quantitative analysis of the percentages of PCNA‐positive cells based on the findings presented in panel (L). N) TUNEL staining of the PDX tumor slices. O) Quantitative analysis of the percentages of TUNEL‐positive tumor cells based on the findings presented in panel N). Data are shown as mean ± SD. *p* values were determined by one‐way ANOVA. in orthotopic models and by a two‐tailed unpaired *t*‐test in PDX models. * *p* < 0.05, ** *p* < 0.01, *** *p* < 0.001, n.s (not significant), *p* > 0.05 compared with the con groups.

Encouraged by the findings that JQ1 treatment can enhance drug penetration and vascularization in PDAC tissues, we explored whether JQ1 treatment can increase the efficacy of antitumor drugs against PDAC. The orthotopic pancreatic cancer model established with primary human a‐PSCs and PANC‐1‐luc pancreatic cancer cells was utilized for this part of the research. The mice were divided into four groups to respectively receive saline, JQ1, GEM, and JQ1+GEM treatments following the scheme shown in **Figure** **6**A. At the end of the treatment, the IVIS system was used to visualize the orthotopic tumors in vivo. As shown in Figure 6B,C, the fluorescence intensities in the saline and JQ1 groups were not significantly different, while that in the GEM group was lower compared with the saline control. Moreover, the JQ1+GEM treatment further decreased the orthotopic tumor fluorescence intensity relative to the GEM alone treatment. The orthotopic tumors were then excised and subjected to IHC and TUNEL assays to determine the percentages of proliferating cell nuclear antigen (PCNA)‐positive and TUNEL‐positive cells for evaluation of tumor cell proliferation and apoptosis, respectively. As exhibited by Figure 6D, the tumors in the JQ1+GEM group were smaller compared to those in the JQ1 or GEM alone groups, and the JQ1+GEM group had significantly lower percentages of PCNA‐positive cells and higher percentages of TUNEL‐positive cells than the JQ1 and GEM groups, indicating that JQ1 treatment augmented the proliferation‐inhibiting and apoptosis‐promoting effects of GEM (Figure 6E–H). Subsequently, we verified these findings in the PDX pancreatic cancer model. The drugs were administered following the scheme presented in Figure 6I. At the end of the treatment, the tumors were excised and weighed. As shown in Figure 6J,K, the JQ1+GEM group exhibited lower tumor weights than the GEM alone group. Additionally, the JQ1+GEM group also had significantly lower percentages of PCNA‐positive cells and higher percentages of TUNEL‐positive cells than the GEM alone group, as revealed by IHC and TUNEL assays (Figure 6L–O). All these findings were consistent with those obtained from the orthotopic pancreatic cancer model. These in vivo findings confirmed that JQ1 treatment enhanced the antitumor efficacy of gemcitabine (GEM) against PDAC.

### 2.7 JQ1 Treatment Enhanced CD8^+^ T cell Infiltration in Tumor Tissues in an Immunocompetent Orthotopic Pancreatic Cancer Mouse Model

**Figure 7:**
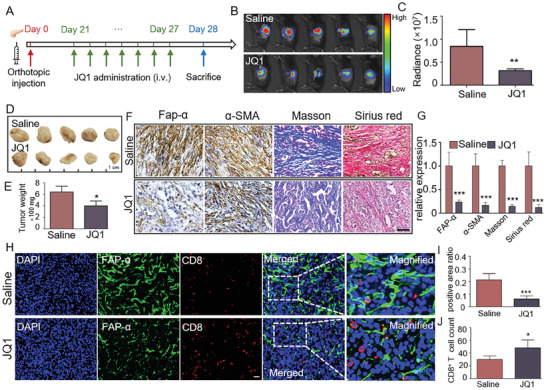
JQ1 treatment enhanced CD8^+^ T cell infiltration into PDAC tumor tissues. A) Scheme of JQ1 treatment in an immunocompetent orthotopic pancreatic cancer mouse model. B) Bioluminescence images for the orthotopic tumor tissues in the Saline and JQ1 groups of mice at the end of the experiment, *n =* 5 for each group. C) Radiance of the orthotopic tumor tissues in the Saline and JQ1 groups of mice. D) Images for dissected orthotopic tumors from the Saline and JQ1 groups of mice. E) Weights of the dissected orthotopic tumors. F) Representative IHC (for FAP‐α and α‐SMA), Masson's trichrome staining, and Sirius Red staining findings in orthotopic tumor slices obtained from the Saline and JQ1 groups of mice. Scale bar, 50 µm. G) Quantification of the signal intensities for the staining analyses displayed in panel (F). H) Representative IF findings for FAP‐α and CD8 in orthotopic tumor slices obtained from the Saline and JQ1 groups of mice. Scale bar, 20 µm. I) Quantitative analysis of the fluorescence signal intensity for FAP‐α. J) Quantitative analysis of the number of infiltrating CD8^+^ T cells. Data are shown as mean ± SD and *p* values were determined by a two‐tailed unpaired *t*‐test. * *p* < 0.05, ** *p* < 0.01, *** *p* < 0.001 compared with the Saline or con groups.

Previous studies have shown that the infiltration of CD8^+^ T cells exhibits a negative correlation with stromal abundance in PDAC tissues.^[^ ^18^ ^]^ In this study, we first tested this conclusion by performing IHC assays for CD8 and α‐SMA in pancreatic cancer tissue. As displayed in Figure S8A–C (Supporting Information), a significant negative correlation existed between the expression level of α‐SMA and the abundance of CD8‐positive cells in the pancreatic cancer tissues. Furthermore, according to the single‐cell sequencing data from TCGA, PDAC tissues having lower stromal scores tended to contain more CD8^+^ T cells (Figure S8D, Supporting Information). These findings confirmed that there is indeed a negative correlation between CD8^+^ T cell infiltration and stromal abundance. Therefore, we hypothesize that JQ1 treatment, which could reduce stromal abundance by reversing the activated phenotype of a‐PSCs, can enhance CD8^+^ T cell infiltration to benefit immunotherapy. To test this hypothesis, we first constructed an immunocompetent orthotopic pancreatic cancer mouse model by injecting a mixture of mPSCs and luciferase‐carrying pancreatic tumor cells (KPC‐luci), which were extracted from pancreatic tumors from KPC mice (LSL‐KrasG12D/^+^; LSL‐Trp53fl/^+^; Pdx1‐Cre) and transfected with luciferase gene, into the pancreas of immunocompetent C57BL/6 mice. Afterward, tumor‐burdened mice were subjected to saline or JQ1 treatment as per the schedule presented in **Figure** **7**A. At the end of the treatment, the mice were observed with the IVIS system to appraise the fluorescence intensity of their tumor tissues. As shown in Figure 7B,C, the fluorescence signal intensity of JQ1‐treated mice was significantly lower than that of saline‐treated mice. Then, the orthotopic tumors were excised and weighed. The results showed that the orthotopic tumors weighed significantly less in the JQ1‐treated group than in the saline‐treated group (Figure 7D,E). We also determined FAP‐α and α‐SMA protein levels with IHC analysis and evaluated collagen deposition with Masson's trichrome and Sirius Red staining. The results indicated that the orthotopic tumors of JQ1‐treated mice had significantly decreased FAP‐α and α‐SMA protein levels and reduced collagen abundance compared with those of saline‐treated mice (Figure 7F,G). Taken together, these data indicated that JQ1 treatment inhibited both tumor growth and tumor stromal fibrosis in the immunocompetent orthotopic pancreatic cancer mouse model. Given that we failed to observe an inhibitory effect of JQ1 on tumor growth in the immunodeficient orthotopic and PDX pancreatic cancer mouse models mentioned above, we speculated that the inhibitory effect of JQ1 on the growth of orthotopic tumors in the immunocompetent mice may be mediated by immune cells. Therefore, we analyzed CD8^+^ T cell infiltration in the orthotopic tumors with IF assays. The results showed that while the proportion of FAP‐α positive cells was significantly decreased, the percentage of CD8‐positive cells was increased by the JQ1 treatment (Figure 7H–J). These results indicated that JQ1 treatment could enhance CD8^+^ T cell infiltration into PDAC tissues by reversing the activation of a‐PSCs

### 2.8 JQ1 Treatment Enhanced the Antitumor Efficacy of IL12 Immunotherapy against PDAC In Vivo

**Figure 8:**
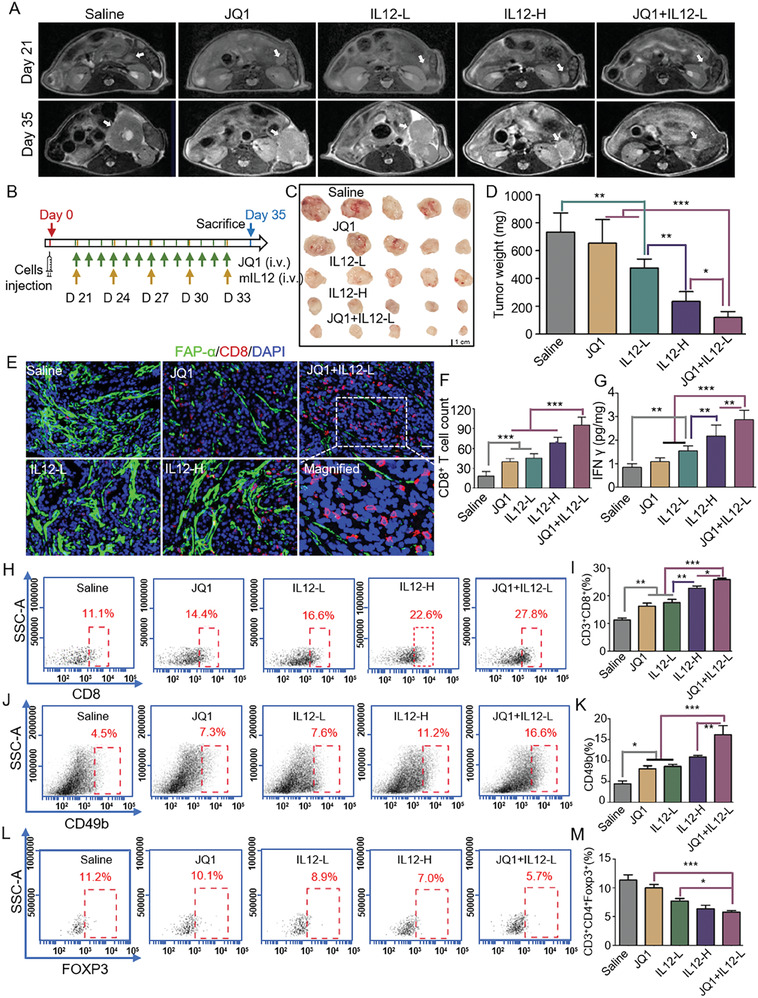
JQ1 treatment enhanced the antitumor efficacy of IL12 immunotherapy against PDAC in vivo. A) Representative MR images for orthotopic tumors of the Saline, JQ1, IL12‐L, IL12‐H, and JQ1+IL12‐L groups of mice captured on Day 21 and Day 35. B) Scheme of JQ1 and different doses of IL12 treatments (or their combinations) in the immunocompetent orthotopic pancreatic cancer mouse model. C) Images for dissected orthotopic tumors from the five groups of mice, *n =* 5 for each group. D) Weights of the dissected orthotopic tumors. E) Representative IF findings for FAP‐α and CD8 in orthotopic tumor sections from the five groups of mice. Scale bar, 20 µm. FAP‐α, green; CD8, red. F) CD8^+^ T cell counts in the orthotopic tumor sections. G) IFNγ concentrations in the orthotopic tumor tissues detected by ELISA. H) Representative flow cytometry scatter plots for CD3^+^CD8^+^ T cells. I) Quantification of intratumoral CD3^+^CD8^+^ T cells for the five treatment groups. J) Representative flow cytometry scatter plots for CD49b^+^ immune cells. K) Quantification of intratumoral CD49b^+^ NK cells for the five treatment groups. L) Representative flow cytometry scatter plots for CD3^+^CD4^+^Foxp3^+^ immune cells. M) Quantification of intratumoral CD3^+^CD4^+^Foxp3^+^ Treg cells for the five treatment groups. *n =* 5 for each group. Data were shown as mean ± SD and *p* values were determined by one‐way ANOVA analysis. * *p* < 0.05, ** *p* < 0.01, *** *p* < 0.001 compared with the Saline or con groups.

CD8^+^ T cells play a fundamental role in immunotherapy. Given that JQ1 treatment could enhance CD8^+^ T cell infiltration into PDAC tissues, we next tested whether JQ1 treatment can benefit immunotherapy. IL12 is a potent immunotherapeutic drug that can activate CD8^+^ T cells and natural killer (NK) cells, enhance their tumor cell‐killing abilities, and interfere with the differentiation of regulatory T (Treg) cells. Multiple clinical trials evaluating the efficacy of IL12 in cancer therapy are underway (34,35). However, the toxicity associated with IL12 limits its application in clinical practice.^[^ ^19^ ^]^ Therefore, we herein sought to enhance the treatment efficacy of IL12 to lower its effective dose in PDAC treatment, thereby reducing its systemic toxicity. We injected the mixture of mPSCs and pancreatic tumor cells derived from KPC mice into the pancreas of immunocompetent C57BL/6 mice to establish the immunocompetent orthotopic pancreatic cancer mouse model and utilized magnetic resonance imaging (MRI) to monitor the initiation of orthotopic tumor formation (**Figure** **8**A). After tumor initiation, the mice were divided into five groups to receive saline, JQ1, IL12‐low dose (IL12‐L: 250 µg k^−1^g), IL12‐high dose (IL12‐H: 500 µg k^−1^g), and JQ1+IL12‐L treatments following the schedule presented in Figure 8B. At the end of the treatment, the orthotopic tumors were observed with MRI and then excised and weighed. The results showed that tumors in the JQ1 group were slightly smaller than those in the saline group; IL12 decreased tumor size in a dose‐dependent manner, while the JQ1+IL12‐L treatment exhibited a significantly better tumor‐inhibiting effect than all the other treatments (all *p*‐Values <0.05) (Figure 8C,D). Then, we determined if these observed tumor‐inhibiting effects were associated with CD8^+^ T cell infiltration inside the tumor tissues by performing IF assays on the excised orthotopic tumor samples to evaluate the protein levels of FAP‐α and CD8. The results revealed that while the JQ1, IL12‐L, and IL12‐H treatments moderately increased the number of tumor‐infiltrating CD8^+^ T cells, the JQ1+IL12‐L group exhibited a significantly higher level of CD8^+^ T cell infiltration compared with all the other treatments (all *p*‐Values <0.05) (Figure 8E,F). To verify these findings, we performed enzyme‐linked immunosorbent assays (ELISA) to evaluate the secretion of IFNγ, one of the most essential effector cytokines released by activated T cells during tumor cell clearance. Consistent with the IF assay findings, the IFNγ secretion by orthotopic tumor tissues was dose‐dependently increased by IL12, and the highest level of IFNγ secretion was observed in the JQ1+IL12‐L group (Figure 8G). To more comprehensively determine the effects of the treatments on immune cell infiltration into the orthotopic tumor tissues, we performed flow cytometry to measure the percentages of CD8^+^ T cells (CD3^+^CD8^+^ cells), NK cells (CD49b^+^ cells) and Treg cells (CD3^+^CD4^+^FOXP3^+^ cells) inside the excised tumor samples. The results for CD8^+^ T cells were consistent with the IF assay findings (Figure 8H,I). Furthermore, the JQ1+IL12‐L combination resulted in the highest percentages of NK cells and the lowest percentages of Treg cells among all the treatments (Figure 8J–M). Taken together, these data suggested that JQ1 disrupted the highly fibrotic pancreatic cancer stromal microenvironment, thereby enhancing IL12 penetration and increasing its antitumor efficacy against PDAC in vivo.

## 3 Discussion

A tumor microenvironment (TME) comprises a highly complex and heterogeneous mixture of resident host cells, tumor cells, tumor‐infiltrating cells, and factors/molecules secreted by them. Many studies have revealed the role of TME in promoting tumor progression including metastasis, chemoresistance, and immunosuppression.^[^ ^20^ ^]^ It is widely believed that TME not only serves as a promoter during cancer development but also can limit the penetration of anticancer drugs by forming biophysical and biochemical barriers in some types of malignancies. This is exactly the case for pancreatic cancer, c malignant digestive disease characterized by a highly fibrotic tumor microenvironment. Abundant studies have revealed that the desmoplastic stroma of pancreatic cancer is a barrier to both chemotherapy and immunotherapy.^[^ ^4^, ^5^, ^21^ ^]^ PDAC is characterized by a dense fibrotic stroma surrounding the tumor cells, which acts as a physical barrier and limits the delivery of therapeutic agents to the tumor. Attenuating fibrosis in pancreatic ductal adenocarcinoma (PDAC) to break the physical barrier has indeed been explored as a strategy to enhance the accessibility of tumor cells to both traditional drugs and nanoparticle drugs.^[^ ^22^ ^]^ According to previous studies and our findings, a‐PSCs play crucial roles in shaping the fibrotic microenvironment of pancreatic cancer.^[^ ^4^, ^5^ ^]^ On the one hand, a‐PSCs produce abundant ECM components, such as collagen, fibronectin, and hyaluronic acid, which, combined with a‐PSCs themselves, constitute the central part of pancreatic tumor tissues. On the other hand, a‐PSCs possess a secretion profile different from that of quiescent PSCs, with the former promoting the progression of pancreatic cancer cells in a paracrine manner.^[^ ^3^, ^4^ ^]^ These complex roles of a‐PSCs in facilitating pancreatic cancer development explain why removing collagen or hyaluronic acid alone could not significantly improve the efficacy of chemotherapy against malignancy.^[^ ^23^ ^]^ Therefore, an increasing number of studies are seeking to understand the molecular mechanism underlying the activation of PSCs and reverse their activated phenotype into a quiescent‐like status, thereby remodeling the tumor microenvironment and facilitating pancreatic cancer drug therapy.^[^ ^3^, ^4^, ^24^ ^]^

Super‐enhancers are a class of cis‐regulatory elements with super‐high transcription‐activating capabilities. The key features that distinguish SEs from typical enhancers include a much larger size (200‐300 bp for TEs compared with ∼20 kbp for SEs), a higher enrichment level of transcription‐activating histone modifications such as H3K27ac and H3K4me, and an increased occupancy of histone acetylation binding proteins (such as BRD4), transcription factors and the mediator complex. These DNA elements can drive many physiological and pathological processes, including tumor cell development. However, to the best of our knowledge, the direct association between SEs in a‐PSCs and the formation of tumor microenvironment has not been documented so far. In this study, we demonstrated that SEs can drive the activation of PSCs in PDAC tissues utilizing a combination of ChIP‐seq, ATAC‐seq, and RNA‐seq techniques. Additionally, we showed that JQ1, a BET inhibitor, suppressed SEs‐mediated gene overexpression in a‐PSCs by reducing BRD4 occupancy, thereby reversing the activated phenotype of a‐PSCs and altering their secretion profile in vitro and in vivo. As a consequence, the treatment markedly reduced the fibrosis degree of PDAC microenvironment and thereby enhanced drug penetration and vascularization. These findings were consistent with the results of an earlier study, which showed that BET inhibitors blocked collagen I production in a‐PSCs but did not pursue the detailed underlying mechanisms.^[^ ^25^ ^]^ Taken together, our data demonstrate that SEs in a‐PSCs play a dominating role in shaping the highly fibrotic PDAC microenvironment, highlighting that SEs can control not only the growth of tumor cells but also the formation of tumor microenvironment in specific malignancies. Notably, although the JQ1 treatment could greatly alleviate tumor stromal fibrosis, it did not significantly affect tumor growth in our two immunodeficient mouse PDAC models.

The highly fibrotic PDAC tumor microenvironment constitutes a major barrier to drug treatments. Many previous studies have demonstrated that drugs possessing the ability to attenuate desmoplasia can enhance the efficacy of chemotherapy or modulate the immune landscape.^[^ ^4^, ^5^ ^]^ Prompted by the findings that JQ1 treatment could greatly reduce stromal fibrosis and increase drug penetration in PDAC tissues, we investigated whether the treatment could serve as a “gate‐opening” strategy to increase the efficacies of chemotherapy and immunotherapy. We first tried to augment the effect of chemotherapy with gemcitabine, the first‐line treatment for PDAC, by applying JQ1 combination treatment. Indeed, the antitumor efficacy of gemcitabine was successfully enhanced by the combination treatment with JQ1. These results were consistent with a previous study showing that the combination of JQ1+gemcitabine was more effective than either drug alone in PDX models.^[^ ^25^ ^]^ Therefore, the clinical significance of this combination warrants further validation. Then we investigated whether JQ1 treatment could benefit immunotherapy. According to previous studies and our results, there is a negative correlation between CD8^+^ T cell infiltration and stromal abundance in PDAC tissues.^[^ ^18^ ^]^ Therefore, we first investigated whether JQ1 treatment could promote CD8^+^ T cell infiltration in PDAC tissues in an immunocompetent orthotopic pancreatic cancer mouse model. The results indicated that JQ1 treatment not only enhanced intratumoral CD8^+^ T cell infiltration but also suppressed orthotopic pancreatic tumor growth in the mice model. In addition to the decreased stromal abundance, these effects can also be attributed to the inhibitory effect of JQ1 on SE‐mediated IL6 overexpression at both mRNA and protein levels in a‐PSCs, because IL6 has been reported as a crucial factor in the formation of an immunosuppressive microenvironment in various types of tumors.^[^ ^26^ ^]^ Given the fundamental role of CD8^+^ T cells in immunotherapy, these data support the possibility of enhancing the efficacy of immunotherapy with JQ1 treatment. IL12 is a very effective immunotherapeutic agent whose clinical translation is largely limited by its systemic toxicity at high doses.^[^ ^27^ ^]^ Therefore, studies have been performed to redesign IL12 to enhance its safety in cancer treatment.^[^ ^28^ ^]^ An alternative strategy to enhance the biosafety of IL12 is to lower its effective dose by increasing its penetration into tumor tissues and enhancing its efficacy. Given that JQ1 treatment can augment both drug penetration and CD8^+^ T cell infiltration into PDAC tissues, we evaluated the immunotherapy efficacy of IL12 at a lower dose of 250 µg k^−1^g in combination with JQ1 in the immunocompetent orthotopic pancreatic cancer mouse model. The findings revealed a better antitumor effect of the combination than any single drug alone. This can be ascribed to the following reason: JQ1 treatment enhanced CD8^+^ T cell infiltration and IL12 penetration. The increased level of IL12 penetration promoted the activation of various types of tumor‐killing immune cells, such as CD8^+^ T cells and NK cells, to form a virtuous cycle that further amplifies tumor elimination effects via a positive feedback mechanism. Taken together, we demonstrated that JQ1 could enhance the efficacies of both chemotherapy with gemcitabine and immunotherapy with a low dose of IL12, revealing its promising potential as a component in combination therapy for pancreatic cancer.

In summary, our study not only elucidates the contribution of SEs in shaping PDAC tumor microenvironment but also highlights that targeting SEs in a‐PSCs by JQ1 may serve as a gate‐opening strategy that improves the efficacies of both chemotherapy and immunotherapy for PDAC by removing tumor stromal barriers.

## 4 Experimental Section

### Clinical Specimen Collection

The collection of specimens from patients was approved by the Ethics Committee of the First Affiliated Hospital of Nanjing Medical University (No. 2021‐SRFA‐026) and all voluntary patients signed informed consent forms. The specimens were collected from patients diagnosed with pancreatic cancer. For immunohistochemistry (IHC) analysis, the surgically excised samples were washed with cold saline, fixed with formalin, and embedded with paraffin. The samples for the construction of the PDX model were washed with saline and transferred into Dulbecco's modified Eagle medium (DMEM, Wisent, Nanjing, China) for cell culturing.

### Immunohistochemistry (IHC) Staining

The paraffin‐embedded sections were deparaffinized in xylenes and rehydrated in an ethanol gradient. After antigen retrieval in a sodium citrate buffer (C1032, Solarbio, Beijing, China) and antigen blocking with 10% goat serum (SL038, Solarbio, Beijing, China), the sections were incubated with antibodies for target proteins overnight at 4 °C in a humidified chamber. Then the sections were incubated with a secondary antibody solution, subjected to chromogenic reactions with diaminobenzidine, and counterstained with hematoxylin. Information for the antibodies used in IHC staining is summarized in supplementary Table S1 (Supporting Information). Image J was used for quantitative analysis of stain‐positive areas.

### Collagen Staining

The collagen deposition in paraffin‐embedded sections was evaluated with Masson's trichrome staining and Sirius Red staining (all reagents from Solarbio, Beijing, China) according to the manufacturer's procedures. The collagen deposited in the tissues was stained blue and red, respectively, in Masson's trichrome staining and Sirius Red staining assays.

### Immunofluorescence (IF) Staining

The cells cultured in confocal dishes or tissue sections were washed by PBS twice and fixed with 4% paraformaldehyde (P1110, Solarbio, Beijing, China) at room temperature for 15 min. After being blocked with PBS containing 10% goat serum (SL038, Solarbio, Beijing, China), the cells or sections were incubated with primary antibodies for target proteins at 4 °C overnight. Then the cells or sections were incubated with fluorescence‐labeled secondary antibodies, followed by nucleus staining with Hoechst (HY‐15559, MedChemExpress, New Jersey, USA). For paraffin‐embedded tissues, the tissues were cut into 5‐µm‐thick sections, deparaffinized in xylene, and rehydrated in an ethanol gradient. Then the sections were incubated with 0.1% Triton X‐100 (T6328, Macklin, Shanghai, Chian) at room temperature for 10 min, blocked with PBS containing 10% goat serum, incubated with primary antibodies at 4 °C overnight, and then incubated with fluorescence‐labeled secondary antibodies for 1 hour at room temperature. After being stained with Hoechst, the sections were observed with fluorescence microscopy. Information for the antibodies used in IF assays is summarized in supplementary Table S1 (Supporting Information).

### Single‐Cell RNA Sequencing and Gene Ontology Analysis

The raw clustered gene‐by‐cell matrixes were downloaded from the Genome Sequence Archive (GSA) database^[^ ^11^ ^]^ under the accession number CRA001160. A total of 24 untreated PDAC and 11 normal pancreatic tissues from Peking Union Medical College Hospital (PUMCH)^[^ ^12^ ^]^ were included in the matrix. The gene‐by‐cell matrixes were filtered to remove cells with <200 genes per cell, >10% mitochondrial genes, <1500 or >25 000 transcripts per cell, and genes expressed in <20 cells using the R package Seurat (v3.2.3). Gene expression levels were normalized with LogNormalize. Afterward, 2000 highly differentially expressed genes were obtained and subjected to principal component analysis (PCA) and uniform manifold approximation and projection (uMAP) to reduce the dimension of data. The identity of each cell cluster was confirmed based on signature genes reported in the literature.^[^ ^13^ ^]^ The expression levels of the selected signature genes in each cluster were summarized into violin plots. The FindMarkers algorithm in Seurat package was applied to identify differentially expressed genes among subpopulations with the criteria of |log_2_ fold change| > 0.5 and *q*‐value < 0.05. Then the top 50 marker genes were subjected to GO analysis using the g: Profiler online tool (https://biit.cs.ut.ee/gprofiler/gost).

### Immune Cell Composition Analysis

The TCGA_PAAD IHC cohort was downloaded from the GDC Data Portal. The ESTIMATE method was applied to calculate the stromal and immune scores of each specimen.^[^ ^29^ ^]^ Next, we performed CIBERSORT with the LM22 matrix to infer immune cell compositions among subgroups with different stromal scores. The default parameters were used.^[^ ^30^ ^]^

### Isolation and Culturing of Pancreatic Stellate Cells (PSCs)

A modified “outgrowth method” was used to isolate human PSCs (h‐PSCs) from tumor tissues excised from pancreatic cancer patients.^[^ ^14b^ ^]^ Briefly, the tumor tissues were cut into blocks with a volume of ≈1 mm^3^ and seeded into wells of cell culture plates. A drop of Matrigel (354234, Corning, New York, USA) was added to each of the tissue blocks, and then the plates were put into an incubator for the solidification of the Matrigel. Then the wells were filled with fresh cell culture medium (DMEM/F12 with 20% fetal bovine serum (FBS)). Two to three days later, the h‐PSCs began to grow out of the tissue blocks.

Mouse PSCs (m‐PSCs) were isolated from the pancreas of C57 mice with the density gradient centrifugation method described in a previous study.^[^ ^31^ ^]^ The mice were anesthetized to obtain their pancreatic tissues on a clean bench. The tissues were cut into pieces and digested into cell suspensions with a buffer containing 0.02% pronase, 0.05% collagenase P, and 0.1% DNAse in Gey's balanced salt solution (GBSS) in a 37 °C incubator for ≈30 min. The cell suspensions were filtered through 150‐µm nylon meshes, resuspended in GBSS with 0.3% BSA, and mixed with the Nycodenz solution (all reagents from Sigma‐Aldrich, Darmstadt, Germany). The mixture was then added on top of the mixture of GBSS with BSA in a centrifuge tube for density gradient centrifugation at 1400 g, 4 °C for 20 min. After the centrifugation, the cells were washed twice with PBS and cultured in DMEM/F12 medium (319‐085, Wisent, Nanjing, China) with 20% FBS (087‐150, Wisent, Nanjing, China).

### ChIP‐seq, ATAC‐seq, and Data Analysis

The ChIP was performed according to a standard procedure. Briefly, 5 × 10^7^ untreated PSCs or those treated with 100 nM JQ1 (MedChemExpress, USA, HY‐13030) for 24 h were crosslinked with 1% paraformaldehyde for 10 min at room temperature. The crosslinking was stopped by incubating the reaction system with 125 nM glycine for 10 min. Afterward, the cells were washed with cold PBS for 3 times and sonicated (15 s × 3 with 30 s intervals) to achieve genomic fragmentation. The cell debris was centrifuged at 12 000 rpm for 10 min at 4 °C to obtain the supernatant, which was incubated with 4 µg of an anti‐H3K27ac antibody (ab4729, Abcam, USA) or an anti‐BRD4 antibody (ab243862, Abcam, USA) or an IgG control overnight at 4 °C. Then the antibody‐chromatin complexes were incubated with Dynabeads Protein G (10007D, Life Technologies, USA) for 2 h at 4 °C. After centrifugation at 1000 rpm for 2 min at 4 °C, the complexes were washed with TSE I (0.1% SDS, 1% Triton X‐100, 2 mM EDTA, 20 mM Tris‐HCl, pH 8.1, 150 mM NaCl), TSE II (0.1% SDS, 1% Triton X‐100, 2 mM EDTA, 20 mM Tris‐HCl, pH 8.1, 500 mM NaCl), buffer III (0.25 m LiCl, 1% NP‐40, 1% deoxycholate, 1 mM EDTA, 10 mM Tris.HCl, pH 8.1) and TE buffer (1 mM EDTA, 10 mM Tris‐HCl, pH 8.0) each in due succession. Finally, the genomic DNA fragments were eluted by an elution buffer (1% SDS, 0.1 m NaHCO3) and subjected to ChIP‐seq or qPCR analyses.

For ATAT‐seq, cells were treated according to the procedure described in a previous work.^[^ ^32^ ^]^ Briefly, 5 × 10^4^ PSCs or PSCs treated with 100 nM JQ1 for 24 h were washed with PBS and collected. Cells were lysed with a lysis buffer to extract their nuclei, which were incubated with Tn5 transposase (FC‐121‐1030, Illumina, USA) for 30 min at 37 °C. The obtained DNA fragments were purified for ATAC‐seq library construction.

The ChIP‐seq, ATAT‐seq, and data analysis were performed by Guangzhou Epibiotek Co., Ltd. using an Illumina HiSeq × 10/Nova platform. For ChIP‐seq analysis, the raw sequencing reads that met the quality control criteria were aligned to a human reference genome using Bowtie 2 Aligner. Model‐Based Analysis of ChIP‐seq (MACs) was used in ChIP‐seq peak calling. Bam files were converted into bigwig files using deepTools and visualized in Integrative Genomics Viewer. H3K27ac ChIP‐seq data for PSCs or JQ1‐treated PSCs were used for super‐enhancer identification by the Rank Ordering of Super‐enhancers (ROSE) algorithm (version 2). Enhancer regions were plotted in an increasing order based on the H3K27ac ChIP‐seq signal. The enhancers above the signal curve inflection point were identified as SEs.

For ATAC‐seq analysis, PeaKDEck was utilized for peak calling ^[^ ^33^ ^]^ and the platform deepTools was applied to normalize the data and to visualize the enrichment of ATAC signals.^[^ ^34^ ^]^

### RNA‐seq and Data Analysis

About 1 × 10^5^ untreated PSCs or those treated with 100 nM JQ1 for 24 h were subjected to total RNA extraction using MiniBEST Universal RNA Extraction Kit (TaKaRa, Japan) according to the manufacturer's instructions. RNA sequencing libraries were prepared using TruSeq Library Prep Kit (Illumina) according to the instructions. The samples were sequenced on a NovaSeq 6000 sequencer (Illumina) to obtain 150‐bp paired‐end reads, which were then aligned with hisat2 to the annotated GTF file of hg38 reference genome within Ensemble (version 91). Expression levels of mRNAs were measured as Fragments Per Kilobase of exon model per Million mapped fragments (FPKM) using HTSeq.

### Deletion of FAP‐α and IL6 Enhancer Fragments with CRISPR‐Cas9 in a‐PSCs

Lentiviruses containing small guide RNAs (sgRNAs) were designed and synthesized by Hanheng Biotechnology Co., Ltd. (Shanghai, China). The targeted sequences of *FAP‐α* and *IL6* enhancers are listed in Table S2 (Supporting Information). Human a‐PSCs were infected with the lentiviruses and selected with puromycin (2 µg mL^−1^) for one week.

### Cell Transfection

The Lipofectamine 3000 reagent (ThermoFisher, USA) was used for the transfection of siBRD4 into a‐PSCs according to the manufacturer's instructions. Briefly, a‐PSCs were cultured in 6‐well plates until the confluency reached 60% when the cells were used for transfection. The siRNA sequences were as follows: negative control (NC), R: UUCUCCGAACGUGUCACGUTT; F: ACGUGACACGUUCGGAGAATT; siBRD4‐1, R: CAGAAGAAACCGAGAUCAUTT; F: AUGAUCUCGGUUUCUUCUGTT; siBRD4‐2, R: CGUAUGAGUCGGAGGAAGATT, F: UCUUCCUCCGACUCAUACGTT. The siRNA was synthesized by Obio technology, in Shanghai, China. For transfection, 4 µg siRNA and 5 µL Lipofectamine 3000 reagent were respectively added into 125 µL Opti‐MEM medium. The two solutions were mixed together and incubated for 15 min at room temperature. Then the mixture was added to a‐PSCs for a 48‐h incubation. Afterward, the cells were collected for western blotting analyses and the supernatant was collected for ELISA.

### Cell Lines

Human pancreatic cancer cell lines were purchased from the Type Culture Collection Committee of the Chinese Academy of Sciences (Shanghai, China) and cultured in DMEM supplemented with 10% FBS at 37 °C. The KPC cell line was isolated from tumors of KPC mice (LSL‐KrasG12D^/+^; LSL‐Trp53fl^/+^; Pdx1‐Cre). These cells were cultured in RPMI 1640 medium with 10% FBS and were transfected with lentiviruses carrying a luciferase reporter gene to construct a luciferase‐expressing KPC cell line (KPC‐Luci), which was used for construction of the orthotopic model. The pancreatic cancer cell line Panc‐1 was also transfected with lentiviruses carrying a luciferase reporter gene to construct Panc‐1‐Luci, which was used in the following animal experiments.

### 3D Cell Culturing

3D tumor spheroids were generated by the hanging drop method according to a procedure reported previously. Briefly, the human pancreatic cancer cell line Panc‐1 was mixed with h‐PSCs at ratio of 1:2 in a cell culture medium containing 0.24% methylcellulose (Sigma‐Aldrich, USA). Twenty microliters of cell suspension containing 20 000 cells was pipetted onto the inverted lid of the cell culture dish, which contained cell culture medium with an appropriate volume of methylcellulose in PBS. Two days later the cells formed tumor spheroids and were subjected to drug treatments. For the drug penetration experiment, the fluorescence dye FITC was added to the cell culture medium containing methylcellulose. Two hours later, the tumor spheroids were washed and transferred into a new dish for observation under a laser scanning confocal microscope. For immunofluorescence assays, the spheroids were washed with PBS and transferred into an optimal cutting temperature (OCT) embedding medium (Sakura, Japan). After being frozen at −20 °C, the blocks were cut into 10‐µm‐thick sections for immunofluorescence staining.

### In Vivo Drug Penetration Experiments

Mice carrying orthotopic pancreatic tumors were treated with JQ1 for one week and then injected with a Cy7 solution (C849890, Macklin, Shanghai, Chian) via the tail vein. Twenty‐four hours later, the orthotopic tumors were excised and observed with an IVIS Spectrum In Vivo Imaging System (PerkinElmer, USA). For evaluation of fluorescent sphere penetration into tumor tissues, the orthotopic tumor‐burdened mice were injected with a fluorescent sphere dilution via tail vein. Twenty‐four hours later, the tumors were excised and made into slices. The slices were then stained with Hoechst and observed by fluorescence microscopy.

### In Vivo Tumor Vessel Imaging

Panc‐1 cells were mixed with a‐PSCs at a ratio of 1:2 and then injected into the pancreas of BALB/C‐nude mice. After one week, the mice with orthotopic tumors were randomly divided into two groups and treated with either saline or JQ1. At the end of the treatment, the mice were anesthetized and given an injection of 70‐kDa FITC‐dextran via the tail vein (150 mg k^−1^g, MedChemExpress, New Jersey, USA). After 30 minutes, the orthotopic tumors were exposed and examined under a multiphoton fluorescence microscope (Leica), and intratumoral vessels were recorded for further analysis. The software AngioTool was utilized to analyze the density of tumor vessels.

### Animal Models

C57BL/6, NOD/SCID, and BALB/C‐nude mice (female, 6–8 weeks age, 20–22 g body weight) were purchased from Vital River Laboratory Animal Technology Co. Ltd. (Beijing, China) and housed in an environment with a 12‐hour light/dark cycle and provided with ad libitum access to food and water. All animal experiments were approved by the Institutional Animal Care and Use Committee of the National Center for Nanoscience and Technology (NO. 20190018). The NOD/SCID mice were used for the construction of the PDX pancreatic cancer model. The specimens were obtained from patients who were diagnosed with PDAC and underwent curative surgical resection. Tumor tissues were excised and transferred to DMEM medium on ice, followed by washing with cold phosphate buffer solution (PBS). The tumors were cut into 2 × 2 × 2 mm^3^ blocks and subcutaneously implanted into the backs of NOD/SCID mice (F1). After six to eight weeks, the PDX‐bearing mice were anesthetized with isoflurane, and the xenografts were excised and implanted into a new batch of NOD/SCID mice (F2). The third generation of PDX models (F3) was constructed similarly to F2. Pathological verification of the tumors was performed by H&E staining to confirm that they were indeed pancreatic cancer tissues. The C57BL/6 mice were injected with KPC cells in the pancreas to build an immunocompetent orthotopic pancreatic cancer model. BALB/C‐nude mice were used to construct another orthotopic pancreatic cancer model by injecting pancreatic cancer cells (Panc‐1‐Luci) mixed with a‐PSCs at ratio of 1:2 into their pancreas. The detailed procedures for constructing orthotopic pancreatic cancer models have been described in our previous work.^[^ ^35^ ^]^ All mice were appropriately anesthetized and then humanely euthanized through cervical dislocation by a highly skilled technician.

### RNA Extraction and qRT‐PCR

Total RNA was extracted from cells using the TRIzol reagent (Thermo Fisher Scientific, USA) according to the manufacturer's instructions. Reverse transcription was performed on the extracted RNA using the QuantiTect Reverse Transcription Kit (Qiagen, Dusseldorf, Germany). Quantitative real‐time PCR (qRT‐PCR) was performed using SYBR Green PCR Master Mix (TOYOBO, Japan) on an ABI7500 real‐time PCR machine (Applied Biosystems, USA) according to the manufacturer's protocol. The primers used are listed in Tables S3 and S4 (Supporting Information).

### Western Blotting Analysis

Cells were collected and lysed in RIPA buffer containing 1% phenylmethanesulfonyl fluoride to prepare total cell lysates. The protein concentration was determined by a bicinchoninic acid assay kit (Thermo Pierce Chemical, USA). Samples of equal protein amounts were resolved by 10% sodium dodecyl sulfate‐polyacrylamide gel electrophoresis (SDS‐PAGE) and then transferred onto polyvinylidene fluoride (PVDF) membranes (Merck Millipore, USA). The membranes were blocked by 5% bovine serum albumin (BSA, Sigma‐Aldrich, USA) for 1 h at room temperature and incubated with primary antibodies overnight at 4 °C. The membranes were then incubated with a corresponding secondary antibody for 1 h at room temperature. Finally, target protein bands were detected by an electrochemiluminescence (ECL) system with an automatic exposure program. The antibody information is listed in supplementary Table S1 (Supporting Information).

### Flow Cytometry

Flow cytometry was used to detect the intratumoral infiltration of different types of immune cells. Tumors were excised from mice and cut into pieces in a digest solution containing protease, DNase, and collagenase. After being digested at 37 °C for ≈30 min, the tissues were filtered with 75‐µm meshes to obtain single‐cell suspensions. The cells in the suspensions were washed with PBS twice and then stained with anti‐CD3‐FITC, anti‐CD4‐APC, anti‐CD8α‐APC, anti‐CD49b‐PE, and anti‐FOXP3‐PE antibodies (BioLegend, USA). Detailed information about the fluorescent antibodies is listed in Table S1 (Supporting Information).

### Enzyme‐Linked Immunosorbent Assay (ELISA)

The Human IL‐6 Precoated ELISA kit (Dakewei, Beijing, China) was used to determine the concentration of IL6 secreted by cells into a medium, as per the manufacturer's instructions. Briefly, 100 µL cell culture medium was added into each well of a 96‐well plate. Then 50 µL of a biotinylated antibody solution was added to the wells and the mixtures were incubated at room temperature for 1 h. After rinsed thrice with a washing buffer (300 µL per well per time), 100 µL of a streptavidin‐horseradish peroxidase solution was added into each well and the 96‐well plate was incubated at room temperature for 20 min. Finally, 3,3′,5,5′‐tetramethylbenzidine (TMB) was added into each well for the chromogenic reaction and a microplate reader was used to detect the optical absorbance of each well at a wavelength of 450 nm.

### Animal Experiments

The orthotopic and PDX pancreatic cancer models were administrated with JQ1 (5 mg k^−1^g) via tail vein according to the procedures shown in Figure 4A,H for the experiments on tumor stroma. For treatments with JQ1, GEM, and their combination, the schemes displayed in Figure 6A,I were followed. The dosage of GEM was 10 mg k^−1^g and it was administrated every other day in the orthotopic and PDX models. In the immunocompetent orthotopic pancreatic cancer model established with C57BL/6 mice, JQ1 (5 mg k^−1^g) was administrated according to the schedule exhibited in Figure 7A. For treatments with JQ1, IL12 (CM39, novoprotein, Suzhou, China), and their combinations, the procedure depicted in Figure 8B was applied. The dosage for JQ1 was 5 mg k^−1^g, while those for IL12 were 250 (for the JQ1+IL12‐L group) and 500 µg k^−1^g (for the JQ1+IL12‐H group). After drug treatments, a magnetic resonance imaging (MRI) machine (Bruker, Germany) was used to observe the orthotopic tumors in the mice.

### Statistical Analysis

All the results were shown as mean ± standard deviation. Data were analyzed by GraphPad Prism 5 using Student's t‐test or one‐way analysis of variance. Differences between groups were considered statistically significant when the two‐sided *p* values were <0.05.

## Conflict of Interest

The authors declare no conflict of interest.

## Author Contributions

Y.W., K.C., G.L., and C.D. contributed equally to this work. Y.W., C.D., and Y.Z. conceived and designed the project. Y.W., K.C., Z.C., and D. Wei performed the experiments. Y.W., D.W., F.L., and C.L. analyzed the data. G.L. and Y.Z. provided suggestions on the project and revised the manuscript. Y.Y., Y.Z., and G.N. supervised the project. Y.W., Y.Z., and G.N. wrote the manuscript.

## Supporting information

Supporting Information

## Data Availability

The data that support the findings of this study are available from the corresponding author upon reasonable request.
