# Correction to “Disruption of Super‐Enhancers in Activated Pancreatic Stellate Cells Facilitates Chemotherapy and Immunotherapy in Pancreatic Cancer”

**DOI:** 10.1002/advs.202507548

**Published:** 2025-05-23

Adv Sci, 2024 Apr;11(16):e2308637. doi: 10.1002/advs.202308637

The original version of this Article contained an error in Figure 1A (immunohistochemical staining for Fibronectin in the Normal group). The error occurred during figure assembly. The corrected figure has now been replaced with the correct one (the first row of the third column). We apologize for this error.


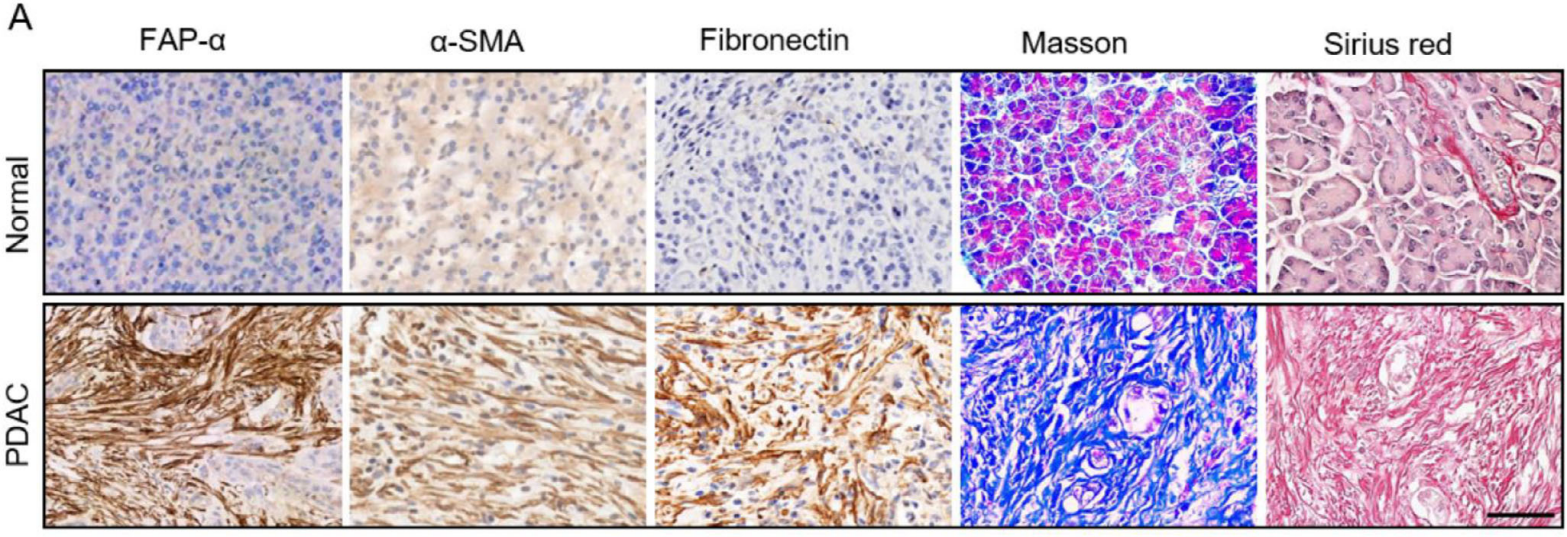


Figure 1A

