# Supplementary material for: Disruption of Super‐Enhancers in Activated Pancreatic Stellate Cells Facilitates Chemotherapy and Immunotherapy in Pancreatic Cancer
Source: Adv Sci (Weinh). 2024 Feb 28;11(16):2308637. doi: 10.1002/advs.202308637 (PMC11040371; doi:10.1002/advs.202308637)
Supplement: Supplementary file 1 — Supporting Information [file ADVS-11-2308637-s001.pdf]

ADVANCED SCIENCE
Open Access

Supporting Information

for *Adv. Sci.*, DOI 10.1002/advs.202308637

Disruption of Super-Enhancers in Activated Pancreatic Stellate Cells Facilitates Chemotherapy and Immunotherapy in Pancreatic Cancer

Yazhou Wang, Kai Chen, Gang Liu, Chong Du, Zhaoxia Cheng, Dan Wei, Fenfen Li, Chen Li, Yinmo Yang, Ying Zhao* and Guangjun Nie**

Supplementary Information

Disruption of Super-enhancers in Activated Pancreatic Stellate Cells Facilitates Chemotherapy and Immunotherapy in Pancreatic Cancer

Yazhou Wang *et al.*

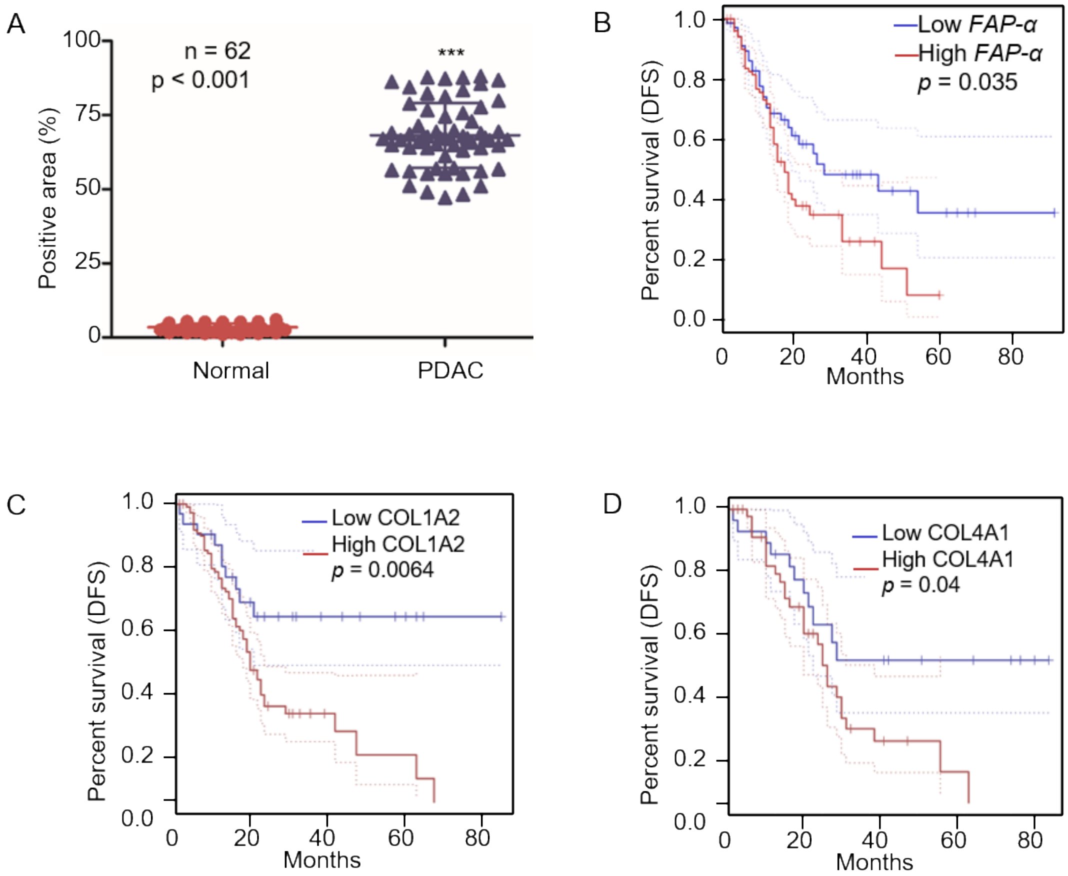

Figure S1. Biomarker analysis of PDAC. (A) Quantitative analysis of FAP-α expression detected by IHC in normal and PDAC tissues, n = 62. **(B-D)** Kaplan-Meier curves of PDAC patients with low or high expression of *FAP-α*, *COL1A2* and *COL1A4*.

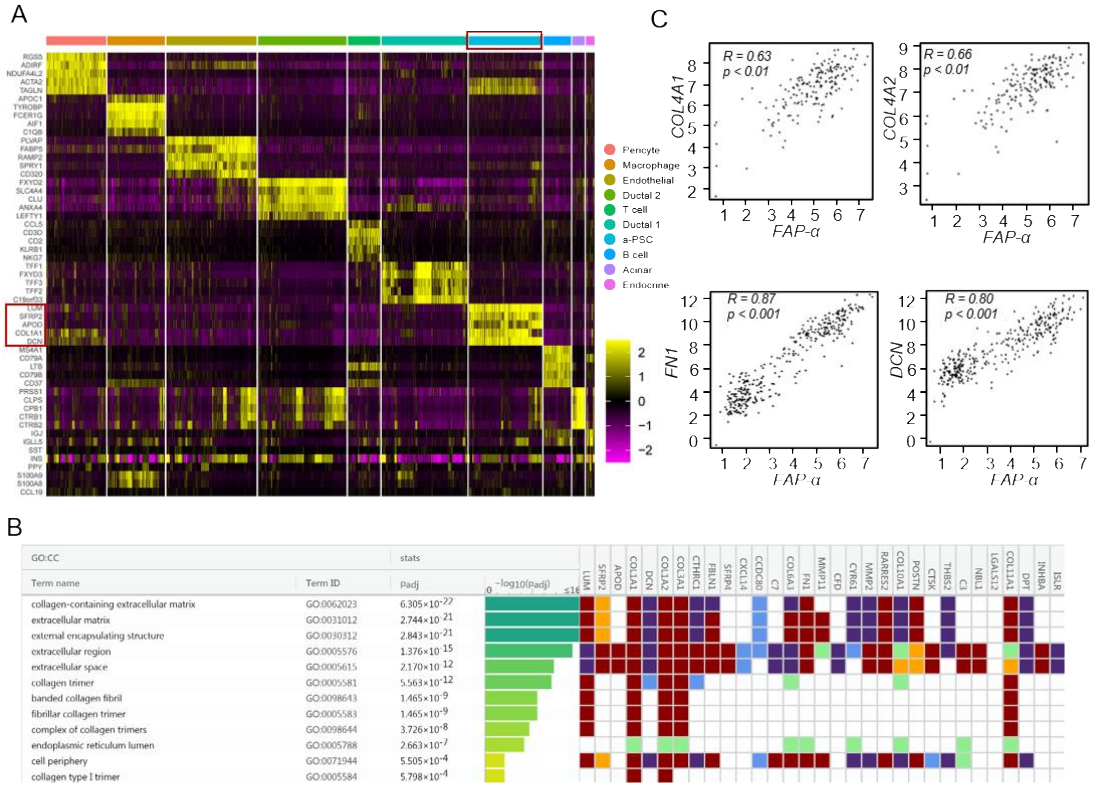

Figure S2. Characteristics of a-PSCs. (A) A heatmap showing the top 5 marker genes for the cell clusters identified by single-cell sequencing. **(B)** Gene ontology analysis showing the representative cellular component terms enriched by the top 50 marker genes in a-PSCs. The colored bars represent the significance level of enrichment. **(C)** Correlations between the expression level of *FAP-α* with those of *COL4A1*, *COL4A2*, *FN1*, and *DCN* in PDAC tissues based on data from TCGA.

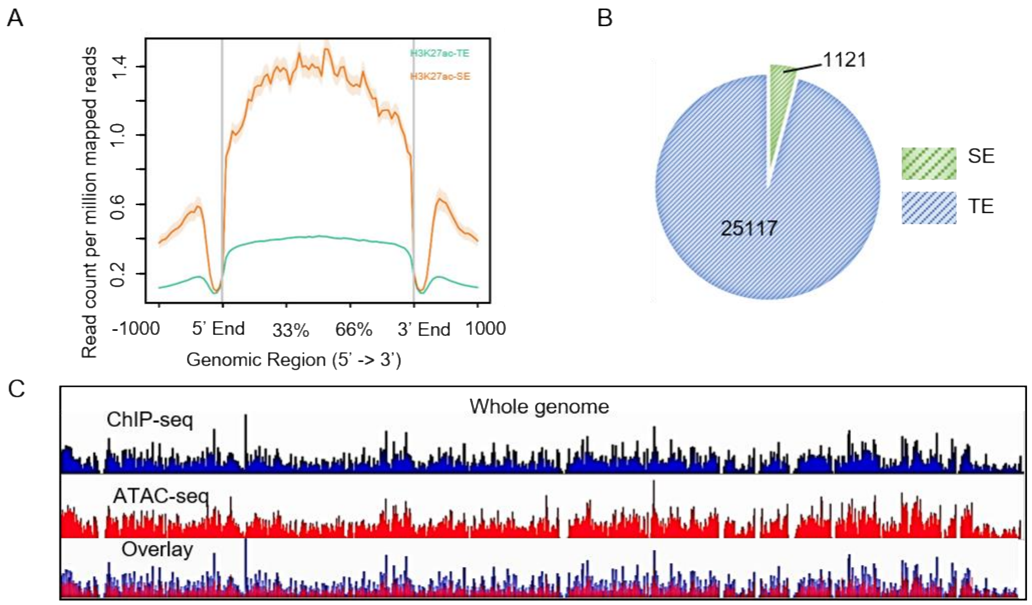

Figure S3. Superen-hancer profile in a-PSCs. (A) Signals for typical enhancers (TEs) and superenhancers (SEs) in a-PSCs. **(B)** A pie chart exhibiting the numbers of TEs and SEs in a-PSCs. **(C)** Whole-genome H3K27ac ChIP-seq and ATAC-seq profiles in a-PSCs visualized by IGV software.

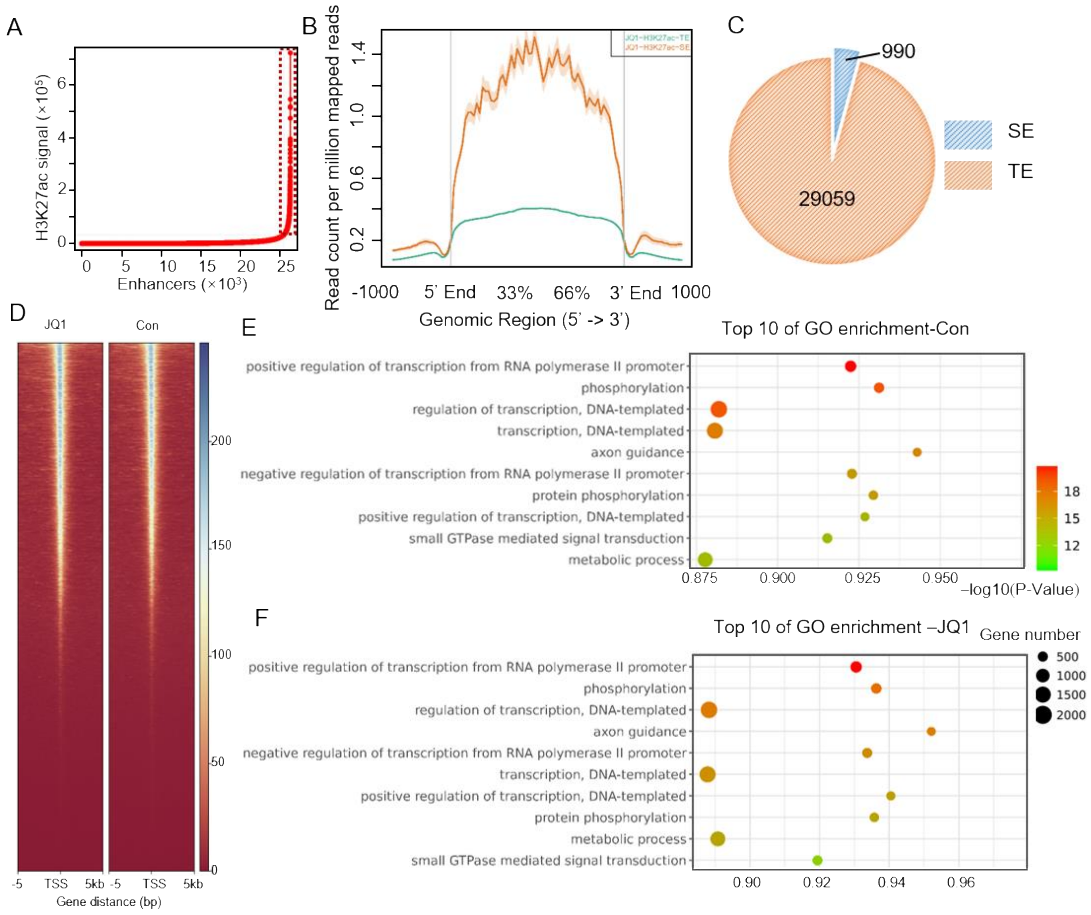

Figure S4. Superen-hancer profile in JQ1-treated a-PSCs. (A) SEs in JQ1-treated a-PSCs identified according to H3K27Ac ChIP-seq signals. Enhancers above the inflection point of the curve (those in the dotted rectangle) were defined as SEs. **(B)** TE and SE signals in JQ1-treated a-PSCs. **(C)** A pie chart exhibiting the numbers of TEs and SEs in a-PSCs. **(D)** ATAC-Seq signals in a-PSCs with and without JQ1 treatment. The intensity of chromatin accessibility is indicated by the color. Peaks are grouped based on K-means clustering. (**E** and **F**) The top 10 GO terms enriched with genes located in open chromatin regions in a-PSCs and JQ1-treated a-PSCs.

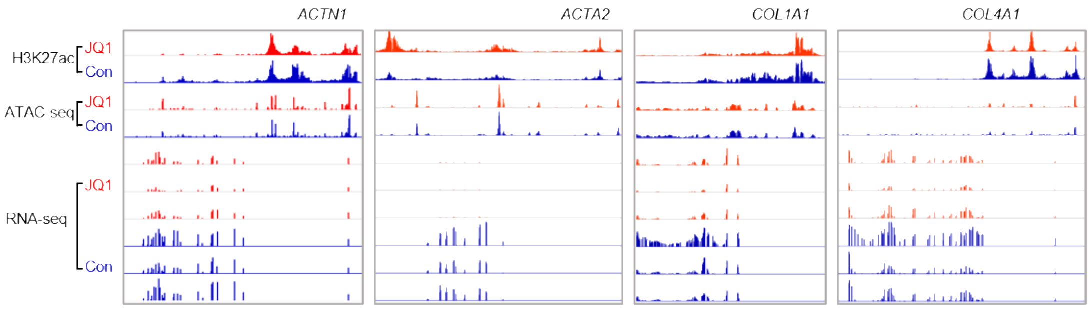

Figure S5. H3K27Ac ChIP-seq, ATAC-seq and RNA-seq profiles for a-PSC-specific genes *ACTN1*, *ACTA2*, *COL1A1* and *COL4A1* visualized by IGV software.

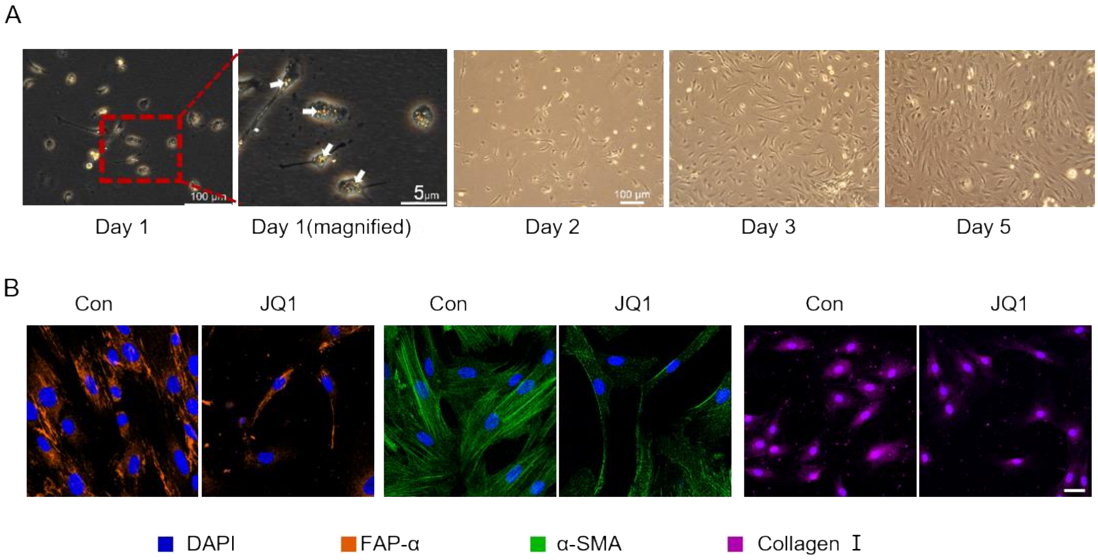

Figure S6. Mouse PSCs culturing. (A) The morphology of mPSCs extracted from the pancreas of C57BL/6 mice imaged at days 1, 2, 3 and 5. **(B)** Representative immunofluorescence images for mPSCs and JQ-1-treated mPSCs stained with anti-FAP-α, α-SMA and collagen I antibodies. Scale bar, 20 μm.

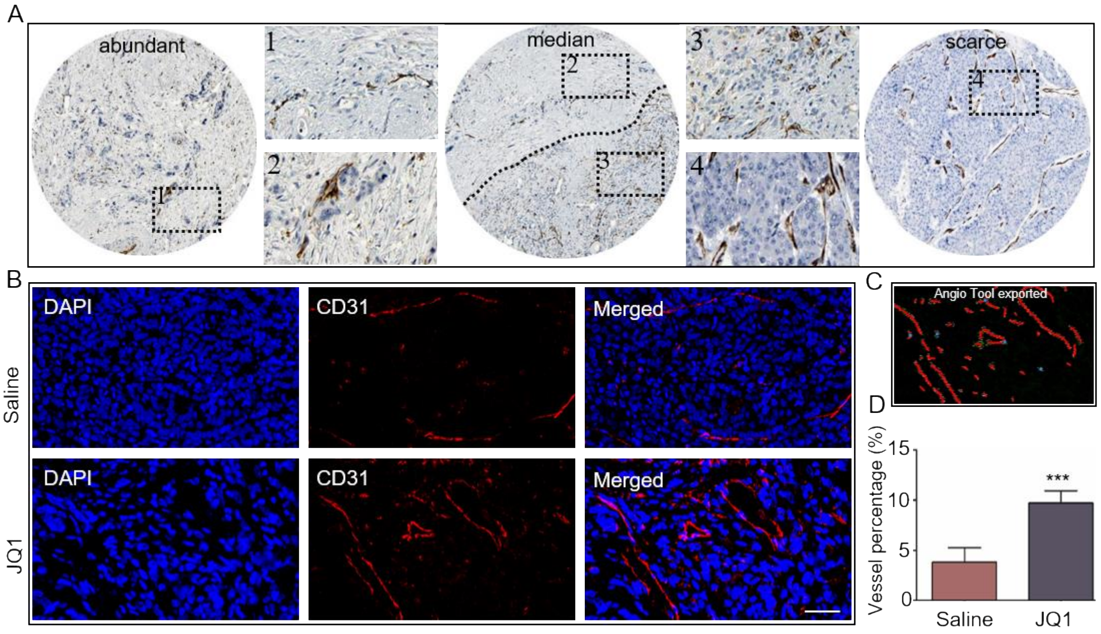

Figure S7. Disruption of SEs promoted vascularization in the patient-derived xenograft (PDX) mouse pancreatic cancer model. **(A)** IHC staining against CD31 in PDAC tissues (data from the Human Protein Atlas database). **(B)** Representative immunofluorescence findings for CD31 in pancreatic cancer tumor slices obtained from PDX mice treated with saline or JQ1. Scale bar, 50 µm. n = 3 for each group. **(C)** A picture exported form AngioTool showing CD31-positive areas in a PDX tumor slice. **(D)** Quantitative analysis of CD31-positive areas in PDX tumors obtained from saline- or JQ1-treated mice. Data are shown as mean ± SD and *p* values were determined by a two-tailed unpaired t-test, *** $p < 0.001$ compared with the con or Saline groups.

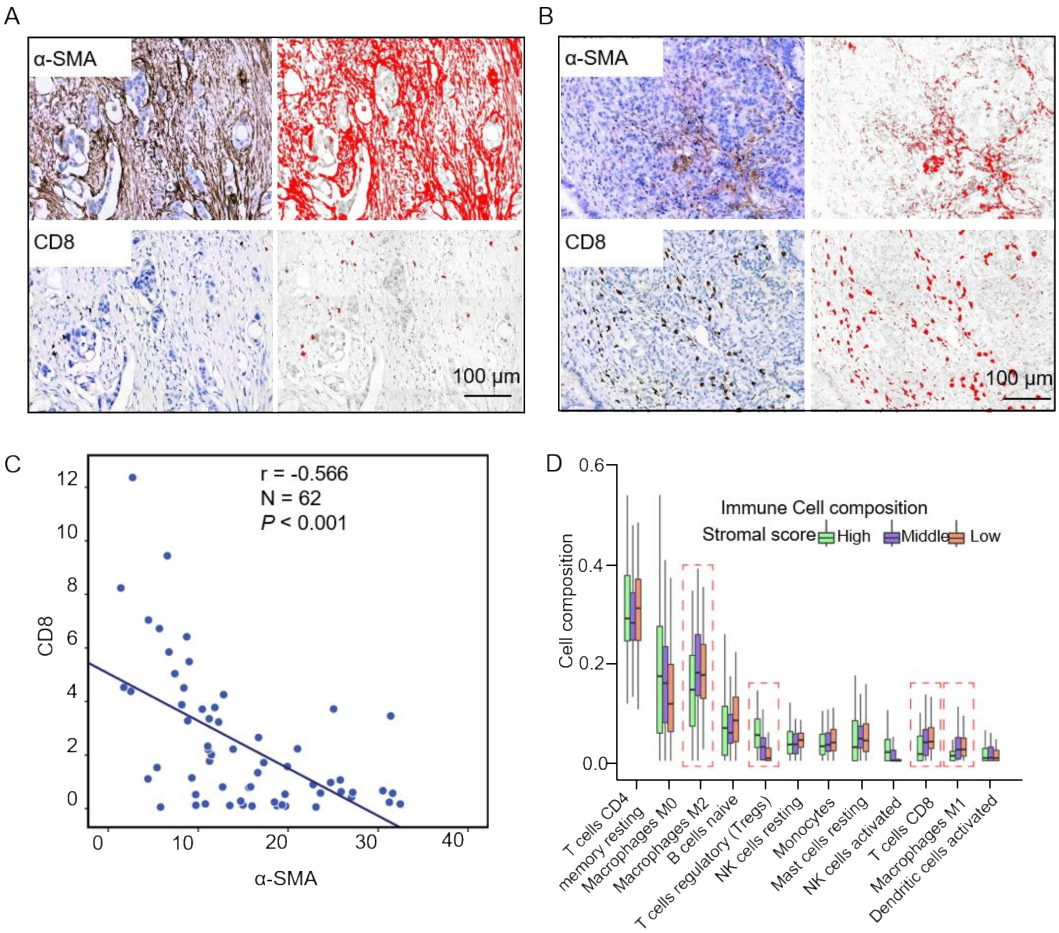

Figure S8. Correlation between stroma abundance and the level of CD8⁺ T cell infiltration in PDAC tissues. (A-B) Abundances of a-PSCs and tumor-infiltrating CD8⁺ T cells, which were respectively reflected by the expression levels of α-SMA and CD8, were detected by IHC in two representative PDAC tissues. The IHC images were analyzed by Image J software. **(C)** The correlation between the expression levels of CD8 and α-SMA in PDAC tissues. **(D)** Stromal scores and immune cell compositions in PDAC tissues exhibited opposite changing trends. Data from TCGA database.

Table S1. Antibody information

Target protein	Application	Manufacturer	Cat. No	Dilution folds
FAP-α	IHC/WB/IF	Abcam	ab207178	1:500/1:1000/1:500
α-SMA	IHC/WB/IF	Abcam	ab7817	1:1000/1:2000/1:1000

Fibronectin	IHC/IF	Abcam	ab2413	1:1000/1:500
Collagen I	IHC/WB/IF	Abcam	ab138492	1:1000/1:2000/1:500
IL6	IF	Abcam	ab233706	1:100
BRD4	WB	Abcam	ab243862	1:1000
CD31	IF	Abcam	ab76533	1:100
PCNA	IHC	Proteintech	10205-2-AP	1:500
CD3	FCM	Biolegend	100204	1:100
CD8	FCM	Biolegend	100712	1:100
CD8	IHC/IF	Abcam	ab237709	1:1000/1:200
Foxp3	FCM	Biolegend	126404	1:50
CD49b	FCM	Biolegend	103515	1:50

Table S2. Target sequences of small guide RNAs used for CRISPR-Cas9 genome editing

Name	Target sequence
FAP-α-SE1	TAAATGGTGAGTAGATCCAC
FAP-α-SE2	TCCGTTACTAGTATTGCAAA
IL6-SE1	TTCAGAACACAGAGACGTCA
IL6-SE2	GGGAGAGTTAGGATGTGCGC
IL6-SE3	TTTACAAACTTCTTACGACT

Table S3. Primer sequences used for qRT-PCR

Gene name	Amplification direction	Sequence
h-FAP-α	Forward	TGGTATAGCAGTGGCTCCAGTCTC
	Reverse	ATCTGCTGTTCCGTGGATGAGAAG
h-ACTA2	Forward	TCGTGCTGGACTCTGGAGATGG
	Reverse	CCACGCTCAGTCAGGATCTTCATG
h-COL1A1	Forward	AAAGATGGACTCAACGGTCTC
	Reverse	AAAGATGGACTCAACGGTCTC
h-IL6	Forward	CACTGGTCTTTTGGAGTTTGAG
	Reverse	GGACTTTTGTACTCATCTGCAC
m-Fap-α	Forward	TTGTTTCGACACCAGCTTTTAG
	Reverse	CCACTTGCCACTTGTAATTTGA
m-Acta2	Forward	GCGTGGCTATTCCTTCGTGACTAC

	Reverse	CGTCAGGCAGTTCGTAGCTCTTC
m-Col1a1	Forward	TGAACGTGGTGTACAAGGTC
	Reverse	CCATCTTTACCAGGAGAACCAT
m-Il6	Forward	CTCCCAACAGACCTGTCTATAC
	Reverse	CCATTGCACAACTCTTTTCTCA

Table S4. Sequences of primers targeting *FAP-α* and *IL6*-associated SEs used for BRD4-ChIP-qPCR

Gene name	Amplification direction	Sequence
FAP-α-SE	Forward	AGAGGTTGTGAGACTTTGCTGTG
	Reverse	ACCCTCCAGCATAACCTCTCTG
IL6-SE	Forward	CACGGCATTCTACCCTGCACTG
	Reverse	AGGCAGGTCACAGGAGACTCTATG
